# f‐Block Element‐Based MOF Thin Films: A Platform for Luminescence, Sensing, and Energy Applications

**DOI:** 10.1002/smll.202514668

**Published:** 2026-01-31

**Authors:** Dong‐Hui Chen, Christof Wöll

**Affiliations:** ^1^ Fujian Key Laboratory of Polymer Materials College of Chemistry and Materials Science Fujian Normal University Fuzhou P. R. China; ^2^ Institute of Functional Interfaces (IFG) Karlsruhe Institute of Technology (KIT) Eggenstein‐Leopoldshafen Germany

**Keywords:** composite MOF particle assembly, electrodeposition, f‐block metal‐organic frameworks, in situ solvothermal growth, layer‐by‐layer, thin film

## Abstract

f‐Block element‐based metal‐organic framework (f‐MOF) thin films, incorporating lanthanides and actinides, have emerged as a promising class of materials due to their unique properties and potential in sensing, luminescence, and energy‐related applications. While MOF powders have been widely explored, many advanced applications—particularly in optics, electronics, and heteroepitaxial integration‐require the controlled architecture and substrate interface that only thin films can provide. This review comprehensively summarizes recent advances in the synthesis, characterization, and functional deployment of f‐MOF thin films, highlighting diverse fabrication techniques such as composite MOF particle assembly, layer‐by‐layer deposition, in situ solvothermal deposition, and electrodeposition. Tabulated data covering polymeric matrices, self‐supporting structures, substrates, and synthesis conditions are included to support comparative analysis. The wide range of demonstrated applications, including luminescence sensing, anti‐counterfeiting, radiation detection, and catalysis, are critically assessed alongside challenges in film stability, scalability, and performance optimization. Finally, future research directions are proposed, emphasizing the need for innovative synthetic strategies, advanced characterization tools, and tailored functional designs to fully exploit the potential of f‐MOF thin films in next‐generation technologies.

## Graphical abstract

f‐Block element–based metal–organic framework (f‐MOF) thin films offer exceptional potential for luminescent, sensing, and energy‐related applications. This review surveys recent progress in their synthesis, characterization, and functional implementation, emphasizing the diversity of available thin‐film fabrication strategies. The convergence of these systems opens a pathway toward multifunctional materials, placing new demands on controlled synthesis and integration for next‐generation applications.

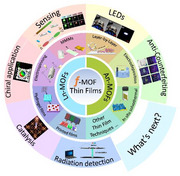

**Abbreviations**
(R)‐ or (S)‐DMPDB: (R)‐ or (S)‐6,6′‐dimethyl‐5,5′‐di(4‐methoxycarbonyl)phenyl‐3,3′‐diiodo‐1,1′‐biphenyl‐2,2′‐diol
1,2,4‐BTC: 1,2,4‐benzenetricarboxylic acid
2,6‐DHTA: 2,6‐Dihydroxyterephthalic acid
2,6‐PDC: Pyridine‐2,6‐dicarboxylate
2‐stp: 2‐sulfonylterephthalate
3,5‐H2bct: 3,5‐bis(3′‐carboxyphenyl)‐1,2,4‐triazole
3,5‐PDA: Pyridine‐3,5‐dicarboxylic acid
3,5‐PZDC: 3,5‐pyrazoledicarboxylic acid
4,4′‐bpy: 4,4′‐bipydine
5AIPA: 5‐aminoisophthalic acid
5‐Tbip: 5‐tert‐butylisophthalic acid
AHBA: 3‐amino‐4‐hydroxybenzoic acid
BABDC: 2,5‐bis(allyloxy)terephthalic acid
BCTPE: (E)‐4,4′‐(1,2‐diphenylethene‐1,2‐diyl)dibenzoic acid
BDC: 1,4‐dicarboxybenzene
bdcbpCl2: 1,1′‐bis(3,5‐dicarboxyphenyl)‐4,4′‐bipyridinium dichloride
BDPO: N,N’ bis(3,5‐dicarboxy‐phenyl)‐oxalamide
BIDC: benzimidazole‐4,7‐dicarboxylic acid
BITD: 5′‐(4,5‐bis(4‐carboxyphenyl)‐1H‐imidazol‐2‐yl)‐ [1,1′:3′,1”‐terphenyl]‐4,4”‐dicarboxylic acid
BPA: bis[3‐(4‐methoxyphenyl)‐propyl]propanedioic acid)
BPDC: biphenyl‐4,4‘‐dicarboxylic acid
BPO: benzoyl peroxide
bpydbH2: 4,4′‐(4,4′‐bipyridine‐2,6‐diyl) dibenzoic acid
BPyDC: 2,2′‐bipyridine‐4,4′‐dicarboxylic acid
BPYDC: 2,2′‐bipyridine‐5,5′‐dicarboxylic
BQDC: 2,2′‐biquinoline‐4,4′‐dicarboxylate
BTA: 1,2,4,5‐benzenetetracarboxylic acid
BTB: 1,3,5‐tri(4‐carboxyphenyl)‐benzene
BTC: 1,3,5‐benzenetrisbenzoic acid
BTDB: 4,4′‐(benzo[c][1,2,5]thiadiazole‐4,7‐diyl)dibenzoic acid
BTEB: 4,4′,4″,4′′′‐benzene‐2,3,5,6‐tetrayl‐tetrabenzoate
BTEC: 1,2,4,5‐benzenetetracarboxylic acid
BTTB: 4,4′,4″,4′′′‐pyrazine‐2,3,5,6‐tetrayl‐tetrabenzoate
CBIA: 5‐(5‐carboxypyridin‐3‐yl)isophthalic acid
CMC‐Na: Carboxymethylcellulose sodium
CS: chitosan
CTP‐COOH: Hexa‐(4‐carboxyl‐phenoxy)‐cyclotriphosphazene
dabco: 1,4‐diazobicyclo[2.2.2]octane)
DCHB: 4′‐di(4‐carboxylphenoxy)hydroxyl‐2,2′‐bipyridyl
Dcam: (1R,3S)‐(+)‐camphorate
DHTA: 2,5‐dihydroxyterephthalic acidic
DOBPDC: 3,3′‐dihydroxy‐4,4′‐biphenyldicarboxylic acid
DPPA: 4‐(2,5‐dicarboxyphenoxy)phthalic acid
DPDF: 1,4‐bis(3′,5′‐dicarboxyphenyl)‐2,3‐difluorobenzene
EMA: ethyl methacrylate
FDA: 2,5‐furandicarboxylic acid
FPTA: 3‐polyfluorobiphenyl‐3’,4,5’‐tricarboxylic acid
H2BTBC: 4′,4′′′‐(benzo[c][1,2,5]thiadiazole‐4,7‐diyl)bis([1,1′‐biphenyl]‐4‐carboxylic acid
H2CPCA: 1‐(4‐carboxyphenyl)‐1H‐pyrazole‐4 carboxylic acid
H2TDA: Thiophene‐2,5‐dicarboxylate
H2TDDA: 4,4‐(1 H ‐1,2,4‐triazole‐3,5‐diyl)dibenzoic acid
H2TNDA: 4,4’‐(1’,3’,3’‐trimethyl‐6‐nitrospiro[chromene‐2,2’‐indoline]‐4’,7’‐diyl)dibenzoic acid
H3ICA: imidazole‐4,5‐dicarboxylic acid
H4BPTC: benzophenone‐3,3′,4,4′‐tetracarboxylate
H4EBTC: 1,1′‐ethynebenzene‐3,3′,5,5′‐tetracarboxylic acid
H4PDIA: 5,5′‐(propane‐1,3‐diylbis(oxy))di‐isophthalic acid
H5DCBA: 3,5‐di(2′,4′‐ dicarboxylphenyl) benozoic acid
HDPB: (1,1′:3′,1′′‐terphenyl)‐3,3′′,5,5′′‐tetracarboxylic acid
Hnpc: 5‐nitro‐2‐pyridinecarboxylic acid
HPAN: Hydrolyzed polyacrylonitrile
Hpbmc: 2‐(pyridine‐2‐yl)‐1H‐benzimidazole‐5‐carboxylic acid
IPIA: 5‐(4‐(imidazol‐1‐yl) phenyl) isophthalic acid
LbL: Layer‐by‐Layer
Lcam: (1S,3R)‐(−)‐camphorate
MC: methylcellulose
MMMs: Mixed‐matrix membranes
MOFs: Metal‐organic frameworks
NaH2SIP: 5‐sulfoisophthalic acid monosodium salt
NDC: naphthalenedicarboxylic acid
NH2‐BDC: 2‐aminoterephthalic acid
Oba: 4,4′‐oxybis(benzoic acid)
PBA: Poly Butyl acrylate
PCL: polycaprolactone
PDA: Polydopamine
PDMS: polydimethylsiloxane
PEBA: polyether block amide
PEG: polyethylene glycol
PEMA: Poly (Ethyl Methacrylate)
PFSA: Perfluorosulfonic acid
Phen: 1,10‐phenanthroline
PI: polyimide
PLA: polylactic acid
PMMA: polymethylmethacrylate
PSF: polysulphone
PTFE: polytetrafluoroethylene
PU: Polyurea
PVA: polyvinyl alcohol
PVA: polyvinyl alcohol
PVDF: polyvinylidene fluoride resin
PVTP: Poly(2‐vinyl terephthalic acid)
RHP: randomly hyper branched polymers
SA: sodium alginate
SAM: self‐assembled monolayer
SDS: sodium dodecyl sulfate
TAA: 1,1,1‐Trifluoroacetylaceton
TATB: 2,4,6‐tris(4‐carboxyphenyl)‐1,3,5‐triazine
TBAPy: 1,3,6,8‐tetrakis(p‐benzoic acid)pyrene
tBrBDC: Tetrabromoterephthalic acid
TCBPA: tris((4‐carboxyl)phenylduryl)amine
tClBDC: 2,3,5,6‐tetrachloroterephthalic acid
TCPP: 5,10,15,20‐(4‐carboxyphenyl)porphyrin
tFBDC: Tetrafluoroterephthalic acid
TGIC: triglycidyl isocyanurate
TPBA: 4‐([2,2′:6′,2″‐terpyridin]‐4′‐yl) benzoic acid
TPE: 4,4′,4′′,4′′′‐(ethene‐1,1,2,2‐tetrayl)‐tetrabenzoic acid
TTA: 2‐Thenoyltrifluoroacetonate

## 1 Introduction

The f‐block elements, encompassing the lanthanides and actinides, have attracted significant attention due to their unique electronic structures, which stem from the progressive filling of the 4f and 5f orbitals [1, 2]. Although both series involve f‐orbital occupation, the 4f orbitals in lanthanides are well‐shielded, leading to predominantly ionic bonding and stable trivalent states. In contrast, the 5f orbitals in actinides are more spatially extended, enabling stronger covalent interactions and a broader range of accessible oxidation states [3]. f‐Block elements exhibit exceptional physicochemical properties, including sharp optical emission [4], high magnetic anisotropy [5], and complex redox behavior [6], providing the basis for a wide range of advanced applications, such as luminescent devices [4], quantum materials [7], catalysis [6], and nuclear energy systems [8]. Despite their potential, conventional f‐block compounds‐such as oxides, phosphates, and halides‐are often limited by poor structural tunability, limited processability, and narrow absorption bands, which constrain their broader applicability in emerging technologies [9].

These limitations can be addressed by metal‐organic frameworks (MOFs) incorporating f‐block elements, which represent a promising class of materials that allow for modular design through the incorporation of organic ligands, enabling precise control over structure and functionality [9, 10]. In contrast to traditional inorganic f‐block compounds, MOFs offer distinct advantages, including enhanced photon absorption capacity, structural adaptability, adjustable porosity, exploiting host‐guest interactions, and improved chemical stability [11, 12]. These characteristics facilitate the precise manipulation of f‐element coordination environments and the fine‐tuning of functional properties [13].

f‐Block elements possess unique optical and electronic properties, including sharp emission lines and long‐lived excited states, making them particularly well‐suited for luminescent applications, such as sensing. In this context, MOFs offer an ideal platform due to their porous architecture, which facilitates efficient analyte diffusion and interaction with the luminescent centers. Their high surface area and inherent selectivity further enable the sensitive detection of specific analytes, including small molecules and ions [14]. In addition, the organic linkers comprising the framework can be switched under external stimuli (in particular light) to add further functionality [15, 16].

The incorporation of lanthanide ions into MOF architectures imparts versatile luminescent behavior and enables the construction of highly responsive molecular sensing frameworks, whereas actinide‐based MOFs demonstrate significant potential in applications such as actinide sequestration, catalysis, sensing, and radiation‐hardened materials [17]. Their superior framework stability, adaptability in host‐guest chemistry, and ability to incorporate multiple functionalities make f‐block element‐based metal‐organic frameworks (f‐MOFs) highly advantageous over traditional f‐block compounds [10, 18].

**FIGURE 1:**
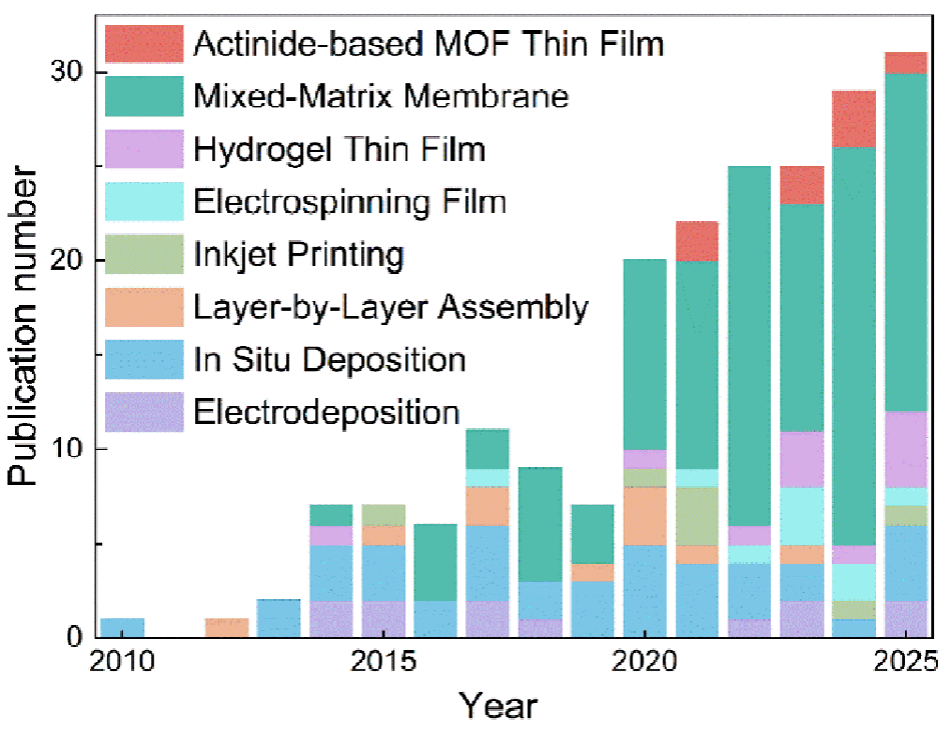
Publications trends of f‐MOF thin films/membranes and their fabrication methods. Publication results were obtained from Web of Science with the topic of all lanthanide/actinide elements & film/membranes. The data was collected on September 14th, 2025.

Building on the unique advantages of f‐MOFs, the fabrication of f‐MOF thin films has emerged as a critical research frontier, driven by the growing demand for device integration and miniaturized functional materials [19]. Precise deposition and controlled crystallization of these MOFs in thin‐film form are essential for applications in luminescent sensing, anti‐counterfeiting, X‐ray imaging, optoelectronics, and selective sorption technologies [20, 21]. Precise deposition further enables the fabrication of hetero‐multilayer f‐MOF thin films, integrating the distinct properties of individual MOF layers to achieve synergistic effects beyond the capabilities of single‐component systems [19, 22]. Although fabricating f‐MOF films is critically important, their deposition onto various substrate types remains a significant challenge. One major difficulty arises from the diverse coordination environments and intrinsic coordination distortions of these metal centers, which lead to structural heterogeneity [10]. Unlike their bulk crystalline counterparts, MOF thin films demand precise control over thickness, uniformity, and surface roughness to ensure film quality and performance [19]. These requirements are particularly stringent in optical applications [23]. The stringent requirements for homogeneity necessitate highly controlled synthesis conditions, including strict regulation of precursor concentrations, deposition parameters, and growth kinetics. Achieving films with reproducible quality and low defect density remains a major challenge, hindering both large‐scale fabrication and practical implementation [22, 24, 25]. To date, various strategies for preparing MOF thin films have been explored, including composite‐MOF‐particle approaches, layer‐by‐layer (LbL) assembly, in situ solvothermal deposition, electrodeposition, and other innovative methods—each offering specific advantages with respect to film thickness control, crystallinity, and compatibility with different substrates. As shown in Figure 1, recent years have witnessed a rapid surge in research on f‐MOF thin films, with a wide variety of potentially applicable materials being fabricated into thin‐film form through diverse synthetic approaches. Advancements in these fabrication techniques have enabled the development of highly ordered, defect‐controlled MOF thin films with enhanced performance and stability.

This review provides a comprehensive analysis of recent advancements concerning f‐MOF thin films, emphasizing their design principles, fabrication techniques, structural characterization, and potential applications in emerging technologies. The discussion goes beyond prior analyses centered on the luminescent applications of lanthanide‐based MOF powders [26, 27, 28]. instead, it summarizes and compares the fabrication methodologies and developmental progress of f‐MOF thin films. Furthermore, for actinide‐based MOFs, this review offers a complete analysis of their thin‐film architectures, offering a critical assessment that extends beyond previous discussions limited to crystallographic studies and bulk‐phase applications [18, 29]. Specifically, it explores various preparation methods for f‐MOF thin films, including composite‐MOF‐particle thin films, LbL assembly, in situ solvothermal deposition, and electrodeposition. Additionally, the review highlights the diverse applications of these thin films, with a particular focus on their use as photonic materials and devices. By drawing parallels between the development of lanthanide‐based MOF thin films and the emerging potential of actinide‐based MOF thin films, this review provides insights into future research directions, anticipating significant progress in the design and utilization of actinide‐MOF thin films for advanced technological applications.

## 2 Fabrication Methods of f‐MOF Thin Film Materials

The fabrication of lanthanide‐ and actinide‐based MOF thin films involves a meticulous process aimed at achieving uniformity, precise thickness control, and multifunctional properties. According to the Hard‐Soft Acid‐Base (HSAB) principle, lanthanide and actinide ions are strong Lewis acids and preferentially coordinate with hard Lewis bases such as carboxylate groups, forming highly stable coordination bonds. As a result, f‐MOFs often exhibit superior structural and hydrolytic stability compared to widely studied d‐block MOFs based on metals such as copper and zinc, positioning them among the most robust metal‐organic frameworks. This enhanced stability enables the application of simpler and more versatile thin‐film fabrication techniques for f‐MOFs. Achieving consistent deposition across large areas while maintaining the desired crystallographic orientation and porosity is a meticulous process that often requires precise tuning of synthesis parameters such as temperature, pressure, and concentration of reactants. Techniques such as composite MOF particle thin film assembly, LbL assembly, MOF particles deposition/in situ solvothermal synthesis, and electrodeposition are commonly employed to construct these films.

### 2.1 Composite f‐MOF Particle Thin Film Assembly

Composite f‐MOF particle thin films constitute a class of secondary processing approaches that involve combining pre‐formed f‐MOF particles with other components to produce various forms of functional materials. Representative techniques include mixed‐matrix membranes (MMMs), hydrogel‐based films, electrospinning, and inkjet printing of MOF‐particle‐doped inks. Presently, this approach stands as the most widely utilized strategy for the fabrication of thin films based on f‐MOFs. The synthesis of MOF particles via hydrothermal or solvothermal methods facilitates their subsequent processing into membranes through relatively straightforward techniques, thereby obviating the need for extensive optimization of film‐forming conditions for well‐characterized MOF materials. This methodology is particularly advantageous in meeting the contemporary demand for scalable and practical film fabrication techniques for f‐MOFs.

Embedding f‐MOF particles into polymers enables the fabrication of flexible, self‐supporting films and membranes. Compared to direct deposition of f‐MOF particles onto substrates, composite thin films with polymeric fillers more effectively address challenges such as continuous film formation and the prevention of pinhole defects that compromise membrane performance. This enhancement is particularly advantageous for applications in gas adsorption and separation, as the MOF fillers facilitates superior control over gas permeability and selectivity, while polymer matrix provides structural support and rapid film shape regulation. Furthermore, f‐MOF‐based films provide enhanced protective capabilities for applications such as temperature sensing, anti‐counterfeiting coatings, and luminescent frequency conversion. The inclusion of polymeric components plays a crucial role in preserving the structural integrity and functional stability of these films under operational conditions.

**FIGURE 2:**
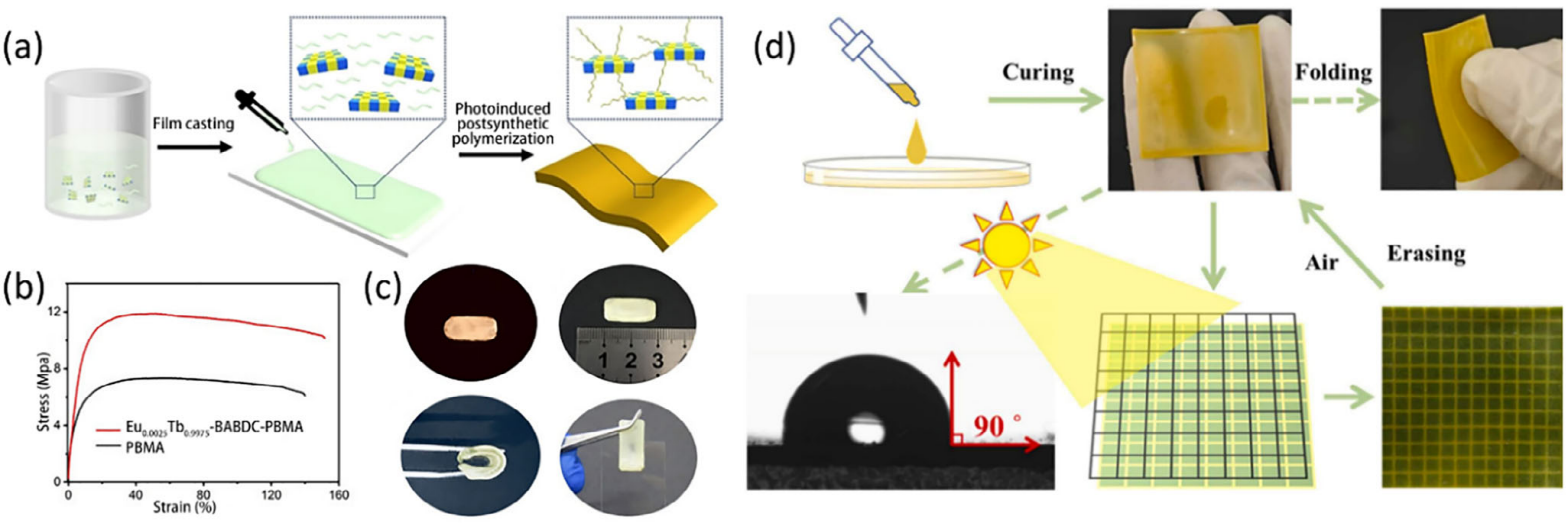
(a) Copolymerization of polymerizable Ln‐BABDC with butyl methacrylate monomers into polyMOF hybrid membrane; (b) The tensile stress–strain curves of the Eu_0.0025_Tb_0.9975_‐BABDC‐PBMA membrane (red) and PBMA (black); (c) Pictures of the Ln‐BABDC‐PBMA membrane under 365 nm UV lamp; (a–c) are reproduced with permission [30]. Copyright 2020, Wiley‐VCH; (d) MMMs method Preparation of the 1‐Eu@PDMS flexible imaging film. Reproduced with permission [31]. Copyright 2025, Elsevier.

Mixed‐matrix membranes (MMMs) constitute a category of composite membranes engineered by incorporating inorganic fillers or organic‐inorganic hybrid materials into a polymeric matrix [32]. As summarized in Figure 1, MMMs currently represent one of the most widely adopted approaches for fabricating f‐MOF‐based thin films. This is primarily due to their favorable processing ease, scalability, versatility, and tunability. As presented in Figure 2, these membranes are commonly fabricated through a casting‐based synthesis approach. This strategy synergistically combines the beneficial properties of the polymeric phase, which offers mechanical flexibility and processability, with those of the dispersed filler phase, which enhances the functional performance of the membrane. The choice of polymer phase is typically determined by a comprehensive evaluation of the polymer's intrinsic properties and the intended application of the MMMs. As summarized in Table 1, polyvinylidene fluoride (PVDF) is often employed in combination with f‐MOFs due to its strong chemical stability, while polyvinyl alcohol (PVA), a highly hydrophilic polymer, is selected for MMMs designed for aqueous‐phase applications. Polydimethylsiloxane (PDMS), owing to its excellent processability, is frequently utilized in applications requiring thin‐film patterning. Poly(methyl methacrylate) (PMMA), with its high optical transparency and ease of processing, is particularly suitable for MMMs leveraging the optical properties of f‐MOFs. Polymers such as Nafion are primarily employed in applications demanding efficient proton transport. Polymers of intrinsic microporosity (PIM‐1), due to their inherent microporous structure, are well‐suited for gas separation or host‐guest recognition. Meanwhile, poly(butyl methacrylate) (PBMA) and similar polymers, despite their lower permeability, exhibit excellent film‐forming properties and processability, making them ideal for MMMs that capitalize on the physical characteristics of f‐MOFs. In recent years, numerous studies have demonstrated that MOF‐based MMMs exhibit outstanding performance in applications such as gas separation and selective sensing, which can be attributed to the distinctive porous structures of MOFs [33]. By leveraging the advantages of MMMs fabrication and the unique physicochemical properties of f‐MOFs, a growing body of research has been dedicated to the development of f‐MOF composite MMMs. These investigations aim to further refine the structural and functional performance of such membranes, particularly for advanced separation and purification applications.

**TABLE 1:** Comparison of f‐MOF thin films by MMMs method.

Polymeric matrix	Metal	Organic ligands	Self‐supporting	Application	Operational medium	Literature
Agarose	Eu	tClBDC	Y	Fluorescent sensing for metronidazole and picric acid	Aqueous (Sensing)	[39]
CS	Ce(IV)	Fumaric acid	Y	CO_2_/N_2_ separation	Gas (Separation)	[40]
Epoxy resin	Ce(III)	BTC	N	Epoxy‐based anti‐corrosion coating	Air	[41]
Epoxy resin	Ce(III)	Imidazole	N	Anti‐corrosion and erosion wear resistance	Aqueous (corrosion)	[42]
Epoxy resin&curing agent	Tb	BTC	N	Smart coating for corrosion detection	Aqueous (anticorrosion)	[43]
Epoxy silane	Eu, Tb	BTC	N	Temperature sensing; White light emission	Air (Sensing)	[44]
Matrimid 5218	Y	BTC	Y	CO_2_ capture	Gas (separation)	[45]
MC	Tb, Gd	BTA	Y	Ln‐containing MOF film preparation method	N/A	[46]
Nafion	Ce(III), Ni(II)	BTC	Y	Electrochemical sensor for bisphenol A	Aqueous (Sensing)	[47]
Nafion	Ce(IV), Zr(IV)	BDC	Y	Proton conductivity	Gas (conductivity)	[48]
PBA&PVA	Ln‐to‐Tb	NH_2_‐BDC	Y	Fluorescence sensors for Th^4+^, UO_2_^2+^, Cr_2_O_7_^2−^, Aldehydes Vapor	Aqueous and vapor (Sensing)	[49]
PBMA	Eu, Tb	BABDC	Y	Fluorescent thermometer	Air (sensing)	[30]
PCL	Eu	BTTB; BTEB	Y	Fluorescence sensors for tetracycline	Aqueous (Sensing)	[50]
PDA	Ce(III)	BDC	Y	Ultrafiltration membranes	Aqueous (Separation)	[51]
PDA	Ce(III)	BTC	N	Superhydrophobic anti‐corrosion coating	Salt Vapor (corrosion)	[52]
PDMS	Eu	BTC	Y	Photoluminescence conversion; anti‐counterfeiting	Air	[53]
PDMS	Eu	NDC	Y	Fluorescence sensors for oxygen	O_2_ gas (Sensing)	[54]
PDMS	Tb, Gd	Alkylated ligand	Y	Dynamic anticounterfeiting and information encryption	Air	[55]
PDMS	Eu, Gd	BdcbpCl_2_	Y	Anti‐counterfeiting; fluorescence sensors for Fe^3+^	Aqueous (Sensing)	[31]
PDMS	Tb	Alkylated ligands	Y	Multi‐level information encryption; anti‐counterfeiting	Air (anticounterfeiting)	[56]
PDMS	Tb	DOBPDC	Y	MOFs‐based flexible scintillator film Imaging	Air (scintillating)	[57]
PDMS	Th(IV)	Hnpc	Y	Photothermal conversion	Air	[58]
PDMS	Eu,Tb	BDC	Y	Anti‐counterfeiting applications	Air	[59]
PEBA	Ho	BTC, BDC	Y	Ho‐MOFs as modifier on membrane transport properties	Aqueous (Separation)	[60]
PEMA	Eu, Tb, Sm, Dy	BTC	Y	Ln‐MOF film preparation method	N/A	[61]
PEMA	Eu,Tb,Sm, Dy, Cd	5‐Tbip	N	Ln^3+^ ion functionalized polymer film preparation	N/A	[62]
PEMA	Eu, Tb, Sm, In	BPYDC	N	White‐Light Emission	Air	[63]
PEMA	Eu(III), Zr(IV)	NH_2_‐BDC	N	Fluorescence sensors for trace water in organic solvents	Organic solvent (Sensing)	[64]
PEMA	Tb, Eu, Sm, Dy, Zn	2‐Stp; 4,4'‐Bpy	N	Fluorescence sensors for Benzene	Urinary (Sensing)	[65]
PEMA	Eu, Tb, Sm, Dy, Mo	PDC	N	White‐Light Emission	Air	[66]
PEMA	Eu, Tb, Sm, Dy, Mo, Cu	BTC	N	White‐Light Emission	Air	[67]
PES	Eu, Dy, Tb	(R)‐ or (S)‐DMPDB	Y	Fluorescence sensors for terpenes and terpenoids	Organic solvent (Sensing)	[36]
PFSA	Ce(III)	BTC	Y	Proton exchange membrane fuel cells	Gas (conductivity)	[68]
PI	Eu,Tb	BTC	N	Luminescent coating film preparation method	N/A	[69]
PIM‐1	Eu(III)	BTC	Y	Chiral recognition and separation	Organic solvent (Separation)	[34]
PLA	Eu,Tb	Ligand containing 12 carboxyl groups	N	Fluorescence sensors for Fe^3+^ ions	Aqueous (Sensing)	[70]
PLA	Tb	Isophthalic acid/phosphazene conjugate	Y	Fluorescence sensors for phenylglyoxylic acid	Urine (Sensing)	[71]
PMMA	Tb	5AIPA	Y	Fluorescence sensors for nitrofuran antibiotics	Aqueous (Sensing)	[72]
PMMA	Nd‐to‐Dy	BTB	Y	Formation and encapsulation of Perovskites in Ln‐MOF film	Air	[73]
PMMA	Tb	BTC	N	Fluorescence sensors for nitroaromatic explosive vapours	Vapor (Sensing)	[74]
PMMA	Tb, Eu, Gd	Nicotinic acid	Y	MOFs‐based flexible scintillator film Imaging	Air (scintillating)	[35]
PMMA	Sm, Eu, Dy, Tb	1,4‐NDC; 2,6‐NDC	Y	X‐ray imaging	Air (scintillating)	[75]
PMMA	Eu, Tb	Phen	Y	Temperature sensing	Air	[76]
PMMA	Eu	BTC	Y	Trichromatic LED modules	Ambient (emission)	[77]
PMMA	Tb	BIDC	N	Fluorescence sensors for benzaldehyde	Vapor (Sensing)	[78]
PMMA	Eu,Tb,Sm,Dy,Yb,Nd,Er,Bi	NaH_2_SIP	Y	Temperature Sensing	Air (Sensing)	[79]
PMMA	Eu, Tb, Gd	2,6‐DHTA	Y	Fluorescence sensors for ethanol	Vapor (Sensing)	[80]
PMMA	Gd, Eu	tClBDC; phen	Y	Fluorescence sensors for nitrophenols	Organic solvent (Sensing)	[81]
PMMA	La, Ce, Dy	H_5_DCBA	Y	Fluorescence sensors for Fe^3+^ and MnO^4−^	Aqueous (Sensing)	[82]
PMMA	Eu, Tb, Sm, Dy	NaH_2_SIP	Y	Anti‐counterfeiting	Air (anticounterfeiting)	[83]
PMMA/PDMS	Tb	BTC	Y	Fluorescence sensors for NO_2_ gas	NO_2_ gas (Sensing)	[84]
PSF	Ce(IV), Zr(IV)	BDC	Y	Dye rejection	Aqueous (Separation)	[85]
PSF & PMMA	Tb	AHBA	Y	Water vapor adsorption and proton conductivity	Vapor (Adsorption)	[86]
PSF/ Matrimid 5218	Eu(II), Tb, Sr(II)	Imidazolate; 4,4'‐bpy	Y	Luminescent mixed‐matrix membranes preparation method	N/A	[87]
PTFE	Eu	3,5‐H_2_bct	Y	Fluorescence sensors for colchicine	Aqueous (Sensing)	[88]
PTFE	Eu, Tb	BTB	Y	Fluorescence sensors for metronidazole and tetracycline	Aqueous (Sensing)	[89]
PTFE	Eu, Tb	H_2_CPCA	Y	Anti‐counterfeiting materials	Air	[90]
PU	Eu(III), Zr(IV)	NH_2_‐BDC	Y	Fluorescence temperature sensing	Air (Sensing)	[91]
PVA	Eu, Tb	CTP‐COOH	Y	Fluorescence sensors for styrene vapor and Fe^3+^ ion	Aqueous/gas (Sensing)	[92]
PVA	Eu, Tb	bpydbH_2_	Y	Proton exchange membranes	Gas (conductivity)	[93]
PVA	Gd	TCPP	Y	Full‐color fluorescence emission	Air	[94]
PVA	Eu, Tb	BTC	Y	Fluorescence sensors for carbamazepine	Aqueous (Sensing)	[95]
PVA	Tb	BTEC	N	Fluorescence sensors for paraquat	Aqueous (Sensing)	[96]
PVA	Eu, Gd, Tb, Dy, Sm	BDPO	Y	Fluorescence sensors for Cr_2_O_7_^2−^, CrO_4_^2−^, Fe^3+^, TNP	Aqueous (Sensing)	[97]
PVA	Pr, Ce	TBAPy	Y	Fluorescent sensing for HCl	Vapor (Sensing)	[98]
PVA	Eu	NH_2_‐BDC; H_2_TDA	Y	Fluorescence sensors for PDC	Serum /aqueous (Sensing)	[99]
PVA	Eu	TPBA	Y	Fluorescence sensors for jatrorrhizine molecule	Aqueous (Sensing)	[100]
PVA	Eu, Tb	BPYDC	Y	Fluorescence sensors for L‐Noradrenaline	Serum (Sensing)	[101]
PVA and hardener	Eu,Tb	H_4_PDIA	Y	Fluorescence sensors for antibiotics	Aqueous (Sensing)	[102]
PVA/PU	Eu	BPyDC	Y	Fluorescence sensors for carcinoid biomarker	Aqueous (Sensing)	[103]
PVDF	Eu(III), Zr(IV)	1,4‐NDC	Y	Fluorescent thermometer	Air (Sensing)	[104]
PVDF	Ce(IV)	BTC	Y	Antithrombotic coating for blood‐contacting medical devices	Blood (Coating)	[105]
PVDF	Eu	Pyromellitic acid	Y	Fluorescence sensors for formaldehyde	Aqueous and gas (Sensing)	[106]
PVDF	Eu	Conjugated polymer ligands	Y	Fluorescence sensors for 1‐hydroxypyrene	Urine (Sensing)	[107]
PVDF	Eu,Tb	IPIA	N	Fluorescence sensors for favipiravir	Serum (Sensing)	[108]
PVDF	Eu	BTDB	Y	Fluorescence sensors for Al^3+^ and Ga^3+^	Organic solvent (Sensing)	[109]
PVDF	Ce(III)	FDA, H_2_TDA, 3,5‐PZDC	Y	Membrane coating in lithium metal batteries	Solid (batteries)	[110]
PVDF	Tb(III),Zr(IV)	BDC	Y	Fluorescence sensors for histamine	Gas (Sensing)	[111]
PVDF	Eu,Tb	DCHB	Y	Fluorescence sensors for 4‐hydroxy‐3‐methoxymandelic acid	Aqueous (Sensing)	[112]
PVDF	Eu	NDC	Y	Fluorescence sensors for antibiotics; Fluorescent thermometer	Aqueous (Sensing)	[113]
PVDF	Eu	HDPB	Y	Fluorescence sensors for hemin and p‐nitrophenol	Organic solvent (Sensing)	[114]
PVDF	Eu, Tb. Zr	Phen; BTC	Y	Temperature sensing	Air	[115]
PVDF	Th(IV)	BITD	Y	Fluorescence sensors for radioiodine species	Organic solvent (Sensing)	[116]
PVDF	Th(IV)	TCBPA	Y	Fluorescence sensors for Cr(VI) anions	Aqueous (Sensing)	[117]
PVDF	Th(IV)	BCTPE	Y	Fluorescence sensors for chromate and dichromate	Aqueous (Sensing)	[118]
PVDF	Th(IV)	BCTPE	Y	Fluorescence sensors for chromate and dichromate	Aqueous (Sensing)	[118]
PVDF & carbon black	Ce(IV)	BTC	N	Electrochemical sensor for dopamine	Aqueous (Sensing)	[119]
PVDF&PVA	Tb	H_2_TDDA	Y	Fluorescence sensors for metronidazole	Aqueous (Sensing)	[120]
PVDF&PVA	Eu,Tb, Na,Zn	FDA	Y	Fluorescent thermometer	Air (Sensing)	[121]
PVDF&Super‐P	Ce(IV)	NH_2_‐BDC	Y	Inhibit the “shuttle effect” in lithium‐sulfur batteries	Solid (batteries)	[122]
PVDF/PCL	Eu	HDPB, phen	Y	Fluorescence sensors for pH and folic acid; visible fingerprint identifying	Aqueous (Sensing)	[123]
PVTP	Tb	BTC	Y	Fluorescence sensors for UO_2_^2+^ in water	Aqueous (Sensing)	[124]
RHP	Eu,Tb	BABDC	Y	Glassy state self‐healing ability; Fluorescent thermometer	Air (Sensing)	[125]
SA	Eu, Tb, Zn	BTC, 2‐ methylimidazole	Y	Fluorescence sensors for acetophenone	Vapor (Sensing)	[126]
TGIC	Tb	NH_2_‐BDC; Phen	N	Fluorescent thermometer	Air (Sensing)	[127]
MC&PEG	Eu	NH_2_‐BDC	Y	Fluorescence sensors for fish freshness	Gas (sensing)	[128]
502 glue	Eu	H_2_TDA	Y	Fluorescence sensors for nitroimidazole antibiotics	Aqueous (Sensing)	[129]

MMMs have emerged as a promising strategy for enhancing chiral separation efficiency, combining the superior selectivity of porous fillers with the processability of polymeric matrices. In the study of Ben, Qiu and coworkers, the integration of chirality‐enriched Eu‐MOFs into a polymer of PIM‐1 matrix exemplifies the advantages of MMMs for enantioselective applications [34]. The chirality‐enriched Eu‐MOFs provide well‐defined chiral channels for host‐guest interactions, while PIM‐1 ensures mechanical stability and facilitates membrane fabrication.

Chen and coworkers fabricated MMMs by incorporating Ln‐MOFs into a polybutyl methacrylate polymer, resulting in a luminescent thermometer with excellent processability, including tailorability, flexibility, and strong adhesion, which originated from its PBMA polymeric matrix [30]. Huang, Zhao and coworkers designed a Ln(III)‐Cu_4_I_4_ heterometallic organic framework exhibiting intense X‐ray absorption and X‐ray‐excited luminescence. By employing the MMM fabrication method, a scintillator film with a high spatial resolution of 12.6 lp/mm was developed through the incorporation of Ln(III)‐Cu_4_I_4_ MOFs into a PMMA matrix [35].

In the work of Han and Cui, MMMs enhanced the enantioselective properties of chiral isostructural lanthanide metal‐organic framework nanosheets (MONs) with the mechanical robustness of polymer matrices, enabling scalable and flexible sensing platforms [36]. This approach enhances stability, facilitates analyte diffusion, and improves recyclability, making MMMs ideal for practical applications in chiral recognition and vapor detection. The fluorescence‐sensing application is examined in greater depth in Section 3.1.

Various f‐MOF materials with specialized functional properties, including applications in anti‐counterfeiting, protective coatings, and photochromic coatings, have been processed into thin films by leveraging the high quality, facile fabrication, and self‐supporting film‐forming capabilities of MMMs. However, the integration of polymeric materials into MMMs can partially obstruct the intrinsic porous channels of MOF particles. This blockage may diminish the selectivity for guest molecules and the response rate of f‐MOF‐based thin films when compared to their powdered forms [37]. Consequently, the functional performance of these films in applications that demand rapid molecular recognition and transport may be somewhat limited. Additionally, the thickness of composite f‐MOF particle‐based films is heavily influenced by several factors, such as the crystal size of the MOF powder precursor, the homogeneity of MOF particle dispersion within the polymer matrix, and the specific coating or deposition method utilized [38]. These limitations pose significant challenges for the fabrication of ultrathin MOF‐based membranes with precise structural control. As such, further research is essential to refine the processing techniques for ultrathin f‐MOF membranes, ensuring enhanced film uniformity, retention of porosity, and optimal functional performance for advanced applications in separation, sensing, and catalysis.

**FIGURE 3:**
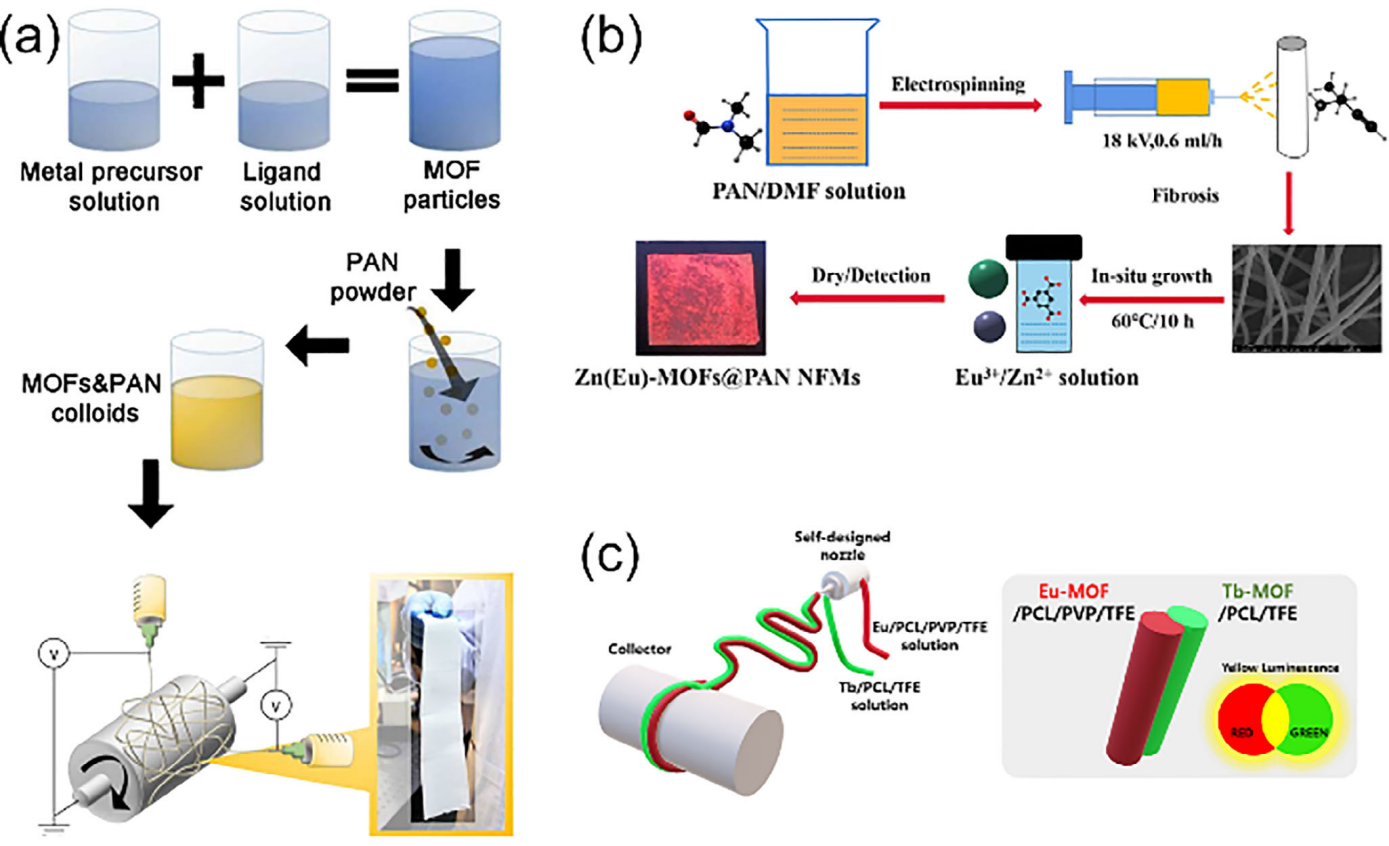
(a) The synthesis process of the MOFs&PAN solution and the experimental setups for multinozzle electrospinning with a drum collector by “MOF‐in‐fiber” strategy; Reproduced with permission [135]. Copyright 2016, Wiley‐VCH; (b) Preparation process of Zn(Eu)‐MOF@PAN nanofibrous membrane by “MOF‐on‐fiber” strategy; Reproduced with permission [136]. Copyright 2023, Elsevier; (c) Schematic illustration of the fabrication procedure of Eu//Tb side‐by‐side nanofibrous membrane. Reproduced with permission [137]. Copyright 2022, Elsevier.

For fabrication of thin films based on composite MOF particles, electrospinning is a widely used technique that offers higher specific interface areas and generally superior mechanical properties compared to other polymer‐based film types. As outlined in previous studies, electrospun MOF‐doped thin films are generally fabricated using one of three approaches: “MOF‐in‐fiber,” “MOF‐on‐fiber,” and “MOF‐seed‐fiber.” [130]. For f‐MOFs, the coordination conditions of f‐orbitals are relatively stringent. As a result, most studies employing electrospinning for f‐MOF thin films have adopted the “MOF‐in‐fiber” and “MOF‐on‐fiber” strategy. (Figure 3) Although membranes produced by electrospinning are generally less suitable for obtaining structures dense enough for purification and separation applications, they are more advantageous for applications leveraging the porous channel characteristics of MOFs. The high specific interface area of electrospinning f‐MOF films has led to promising performance in fields such as fluorescence sensing and highly efficient heterogeneous catalysis. As illustrated in Table 2, polyacrylonitrile (PAN) is the preferred choice for most electrospun membranes due to its semi‐crystalline nature, high solubility in solvents such as N,N‐dimethylformamide (DMF) and dimethyl sulfoxide (DMSO), and structural stability during electrospinning. PAN also boasts high commercial maturity, with its exceptional mechanical strength and thermal stability being well‐documented in the literature. The inaugural synthesis of f‐MOF films via the electrospinning technique was documented by Wang and coworkers [131]. In their study, a highly stable nano‐sized Ce‐MOF was successfully engineered into an effective coating catalyst for Knoevenagel condensation reactions. This achievement was realized through strategic defect engineering and the application of electrospinning fabrication methods. Furthermore, Zhu and coworkers highlighted the rapid, efficient, and real‐time detection capabilities of electrospun Ln‐MOF films, alongside their superior recyclability, underscoring the potential of these materials in practical applications [132, 133, 134].

**TABLE 2:** electrospinning method.

Polymeric matrix	Strategies	Metal	Organic ligands	Application	Operational medium	Literature
PAN	“MOF‐in‐fiber”	Ce(III)	BTB	Coating catalyst	Organic solvent (Catalysis)	[131]
PAN	“MOF‐on‐fiber”	Ce(IV)	BDC	Antibacterial adsorbent	Wastewater (Separation)	[138]
PAN	“MOF‐in‐fiber”	Tb	BTC	Phosphate capture	Aqueous (capture)	[139]
PAN	“MOF‐on‐fiber”	Eu,Zn	BTC	Fluorescence sensors for nitrobenzene. 4‐nitrophenol, benzaldehyde, Fe^3+^ ions	Organic solvent (Sensing)	[136]
PAN	“MOF‐in‐fiber”	Eu,Tb	FPTA	Fluorescence sensors for 2,6‐pyridinedicarboxylic acid	Aqueous (Sensing)	[134]
PAN	“MOF‐in‐fiber”	Tb, Gd	BPA	Fluorescence sensors for adenosine triphosphate	Urine (Sensing)	[132]
PAN	“MOF‐in‐fiber”	Eu, Tb, Gd	DPDF	Fluorescence sensors for dopamine	Aqueous (Sensing)	[133]
PCL/PVP	“MOF‐in‐fiber”	Eu,Tb	BTC	Fluorescence sensors for Fe^3+^ and Cu^2+^	Aqueous (Sensing)	[137]
PMMA /PVP	“MOF‐in‐fiber”	Eu	BDC	Fluorescence sensors for uric acid	Urine (Sensing)	[140]

**FIGURE 4:**
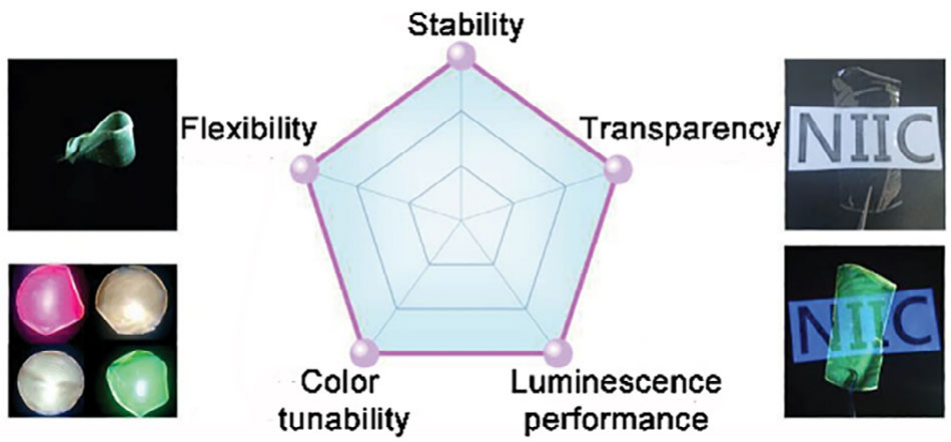
Five advantages of composite hydrogel film materials. Reproduced with permission [146]. Copyright 2024, Wiley‐VCH.

Hydrogel films, composed of three‐dimensional networks of hydrophilic polymeric chains with a high‐water content (90–99%) [141, 142]. offer significant advantages such as edibility and cost‐effectiveness, making them attractive for biomedical and sensing applications, as shown in Figure 4. Their inherent biocompatibility, low immunogenicity, minimal cytotoxicity, and tunable physicochemical properties further enhance their utility in various fields [143]. As summarized in Table 3, natural polysaccharides such as sodium alginate, chitosan, and agarose exhibit excellent biocompatibility and moderate adhesiveness, along with favorable biodegradability. These properties make their composites with f‐MOFs particularly suitable for biomarker monitoring applications. In contrast, sodium carboxymethyl cellulose (CMC‐Na), while cost‐effective and easily processable, demonstrates hygroscopic tendencies that may compromise film integrity. Nevertheless, its composites with f‐MOFs show promising potential for gas sensing applications. Incorporating f‐MOFs into hydrogel films has demonstrated promising potential in biosensing applications due to the synergistic combination of the structural versatility of MOFs and the functional adaptability of hydrogels. This approach not only facilitates the preservation of the physical and chemical properties of f‐MOF particles but also enables the development of novel thin‐film materials with performance comparable to their powder counterparts. Thus, hydrogel‐based MOF films represent an emerging class of functional materials with broad application prospects in advanced sensing technologies.

**TABLE 3:** Comparison of f‐MOF hydrogel thin films.

Polymeric matrix	Metal	Organic ligands	Self‐supporting	Application	Operational medium	Literature
Agar or Gelatine	Eu	BDC	Y	Fluorescence sensors for the condition of the materials	Aqueous (Sensing)	[147]
Agarose	Eu,Tb	CBIA	N	Fluorescence sensors for 2,6‐dipicolinic acid	Aqueous (Sensing)	[81]
Al–O or Ti–O network	Eu,Tb, Al	TAA; TTA; PDA	N	White Light emission	Air	[148]
Bacterial Cellulose	Eu	BTC	Y	Fluorescence sensors for ammonia gas	Vapor (Sensing)	[149]
Carrageenan	Eu,Tb	DPPA	Y	Fluorescence sensors for OFX in chicken meat; Anti‐counterfeiting; Afterglow tunability	Vapor (Sensing)	[146]
Cassava	Ce(III)	5AIPA	Y	Food packaging biopolymer films	Air	[150]
Chitosan	Ce(III)	BTC	N	Electrochemical sensor for tryptophan	Serum (Sensing)	[151]
CMC‐Na	Eu, Tb, Hf(IV)	BTEC	N	Fluorescence sensors for pyrethroid	Vapor (sensing)	[144]
CMC‐Na	Eu	BDC	Y	Fluorescence sensors for fresh‐cut fruit freshness	Vapor (Sensing)	[152]
Sodium alginate	Eu, Zn	BTC	Y	Fluorescent sensor for amino acids (Aspartic acid, arginine)	Serum (Sensing)	[145]
Sodium alginate	Eu, Tb, Ce(III)	H_4_EBTC	N	Fluorescence sensors for vitamin B6	Aqueous (Sensing)	[153]
Sodium alginate	Eu, Tb, Zr	BDC, NDC	Y	Film‐based array sensor for nitrophenol isomers	Aqueous (Sensing)	[154]
Sodium alginate	Eu(III)	BTB	Y	Permeability and selectivity for ethanol dehydration	Organic solvent (Purification)	[155]

Yan and coworkers demonstrated a two‐dimensional Ln‐MOF hydrogel film for the monitoring of pyrethroid biomarkers and amino acid, enabling the simultaneous detection of exposure time and extent with high sensitivity and selectivity. The incorporation of hydrogel in sensor film construction enhances portability, facilitates visual detection, and preserves the structural stability and luminescent properties of Ln‐MOFs [144, 145]. In 2024, Fedin and colleagues introduced Ln‐MOF‐based hydrogel films with tunable luminescence and afterglow properties, achieving highly selective and sensitive detection of ofloxacin (OFX) at ultralow concentrations (1.1 × 10^−^^9^ M) while also demonstrating advanced anti‐counterfeiting applications. The use of hydrogel films in the fabrication of Ln‐MOF‐based materials is particularly advantageous, as hydrogels provide a flexible, biocompatible, and water‐stable matrix that maintains the physical and chemical integrity of Ln‐MOF particles while broadening their applicability in sensing and security technologies. Furthermore, hydrogel films enable the efficient production of portable, visually interpretable luminescent materials, making them highly suitable for on‐site antibiotic detection in food safety and environmental monitoring applications [146].

**FIGURE 5:**
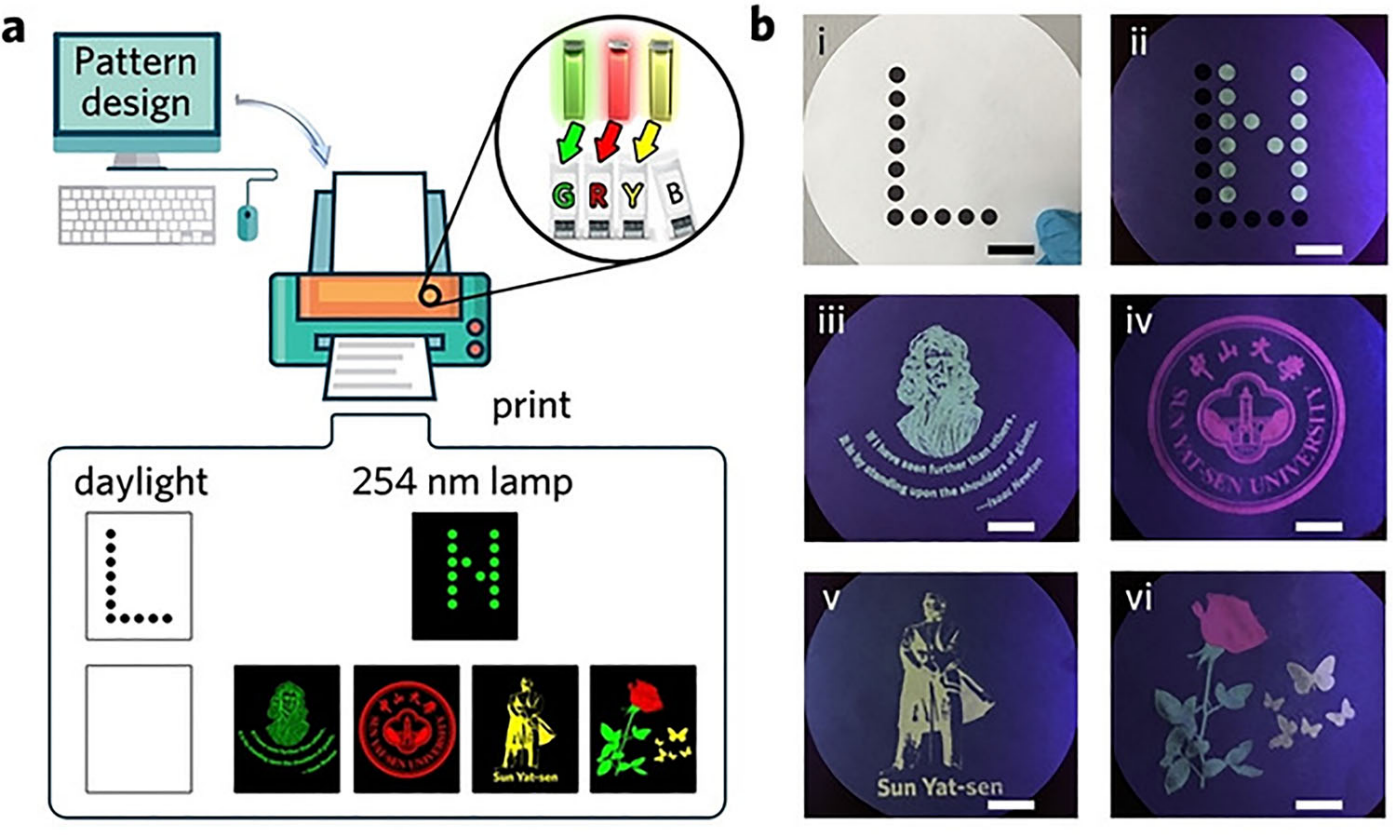
(a) Schematic illustration of pattern encryption in the spatial dimension via pattern design and inkjet printing process with green, red, and yellow security inks; b) Photographs under daylight or 254 nm lamp irradiation. Scale bars in (b) represent 2 cm. Reproduced with permission [157]. Copyright 2020, Wiley‐VCH.

Incorporating f‐MOFs into printable inks and their subsequent deposition onto substrates has gained significant attention as a promising method for the fabrication of composite particle thin films. By capitalizing on the stable visible‐light emission characteristics of f‐MOFs, it is possible to develop luminescent inks that can be employed in inkjet printing to create customizable two‐dimensional patterns. Owing to the favorable chemical stability imparted by high‐charge lanthanide ions coordinated with organic ligands, f‐MOFs can be dispersed under a variety of conditions for the preparation of luminescent inks, as summarized in Table 4. The selection of dispersants and surfactants is critical, requiring not only efficient wetting of f‐MOF particles within a short period but also the maintenance of colloidal stability. Given the oxophilic nature of f‐block ions and the prevalence of carboxylate‐based linkers in these MOFs, alcoholic dispersants have emerged as the primary solvent choice in current practices. In 2015, Júnior and coworkers pioneered the use of inkjet printing for the fabrication of Ln‐MOF thin films [156]. They dispersed Eu_2_(Mellitate) and Tb_2_(Mellitate) particles into ink formulations and successfully printed patterned films onto plastic foils and paper using a conventional inkjet printer. Subsequently, Liang and coworkers developed a series of lanthanide‐doped Y‐BTC MOFs (Tb^3^^+^, Eu^3^^+^, Sm^3^^+^) by introducing an energy transfer (ET) manipulation strategy based on host differential sensitization. This approach enabled large‐scale color tunability from green to red and adjustable lifetimes of green emission within a range of 300–600 µs. As illustrated in Figure 5, the preparation of f‐MOFs into inkjet printing facilitated the realization of luminescence lifetime imaging and time‐gated imaging concepts. These MOFs, characterized by their efficiency, stability, and tunable optical properties, were further employed in the development of security inks [157]. In 2024, Li and colleagues reported embedding spiropyran‐like photoactive molecules into the cavities of Ln‐MOFs, creating dynamically photo‐responsive materials (Ln‐MOFs@SP). These materials were dispersed into inks and utilized for the fabrication of invisible two‐dimensional (2D) patterns. This method enabled multi‐channel, cross‐disciplinary information encryption and decryption, achieving applications such as invisible 2D codes, fluorescent 2D codes, and fluorescent 3D codes through the construction of multicolour modular 2D codes [158].

**TABLE 4:** Comparison of f‐MOFs into printable inks.

Dispersant	Substrate	Surfactant	Metal	Organic ligands	Self‐ supporting	Application	Operational medium	Literature
Ethanol	PET film /Paper	N/A	Eu, Tb, Gd, Nd	Mellitate	N	Anti‐Counterfeiting	Air	[156]
Ethanol & glycol & glycerin & diethylene glycol	Filter paper	SDS	Eu, Tb, Sm, Y	BTC	N	Luminescence lifetime imaging and time‐gated imaging	Air (imaging)	[157]
DMSO & ethanol & ethylene glycol	Paper /PET foil	N/A	Tb	BTC	N	Fluorescence sensors for ammonia	Vapor (Sensing)	[159]
PEG1788	Paper	SDS	Eu, Tb, Zn	H3ICA	N	Photo‐switching; white light‐emitting nanopapers	Air	[160]
Ethanol	Paper	N/A	Eu, Tb, La	4,5‐dichlorophthalic acid	N	Anti‐counterfeiting	Air (anticounterfeiting)	[161]
Ethanol &PVA &PEG Or PU tactile dumb oil	Paper	N/A	Eu, Tb, Gd	BTC	N	Information storage and encryption	Air (anticounterfeiting)	[158]
Ethanol	Paper	N/A	Eu, Tb, Sm	Pyridine‐2,6‐dicarboxamide‐based linker	N	Fluorescence sensors for nitrobenzene & 4‐nitrophenol;Anti‐Counterfeiting	Aqueous (Sensing)	[162]

### 2.2 Layer‐by‐Layer (LbL) Assembly

**FIGURE 6:**
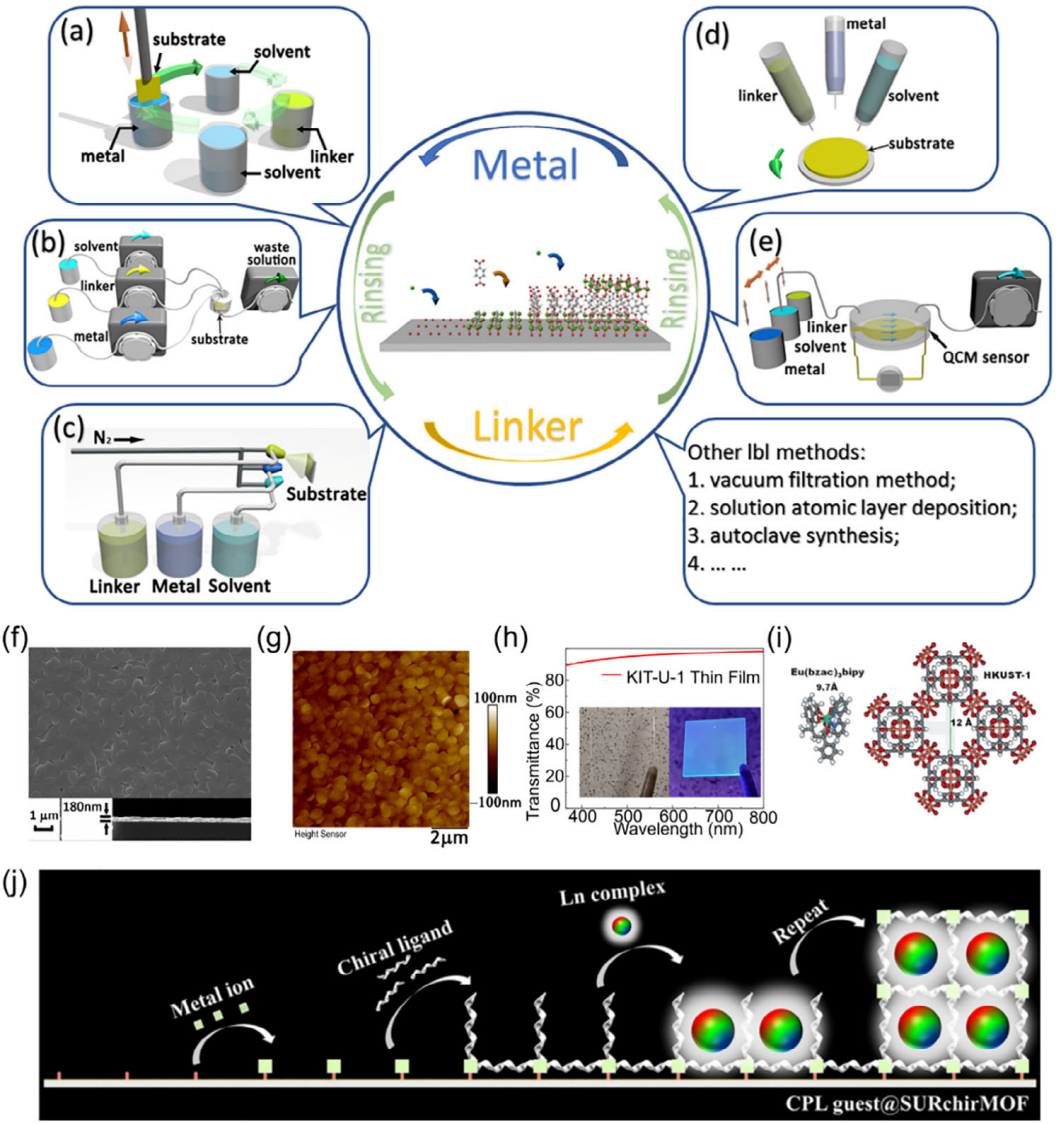
Schematic illustration of the layer‐by‐layer assembly of SURMOFs: (a) dipping method, (b) pumping method, (c) spray method, (d) spin coating method, and (e) LbL synthesis on QCM system. Reproduced with permission [19]. Copyright 2023, American Institute of Physics; (f) SEM image of f‐thin film (KIT‐U‐1) by LbL assembly from a top view (top) and cross section (bottom); (g) AFM image of KIT‐U‐1; (h) UV–visible transmittance spectra of KIT‐U‐1 thin film in visible light range, inset photographs of KIT‐U‐1 under sunlight (left) and 285 nm ultraviolet light (right); Reproduced with permission [170]. Copyright 2023, Wiley‐VCH; (i) size of the Eu(bzac)_3_bipy complexes and pore size estimation of Cu_3_(btc)_2_.xH_2_O‐MOF; Reproduced with permission [173]. Copyright 2012, Wiley‐VCH; (j) Ln‐complex loaded chirMOF thin film prepared by LbL encapsulation method for CPL property. Reproduced with permission [175]. Copyright 2022, Springer.

The LbL assembly of MOF thin films, in particular, allows for the sequential deposition of f‐block metal ions and organic linkers, enabling precise control of f‐MOFs over film composition and thickness at the molecular level [162]. The ability to fabricate MOF thin films via LbL deposition represents a significant advancement in the fabrication of highly ordered and functional materials. As shown in Figure 6a–e, this technique allows for precise control over film thickness and composition, enabling the creation of ultra‐thin MOF films with tailored properties [19]. One major advantage of LbL growth compared to using dispersed powders is that it is not constrained by particle size, allowing the fabrication of highly uniform films with minimal thickness. Additionally, in several cases it has been demonstrated that LbL situ growth on well‐defined substrates results in fewer defects, enhancing the structural integrity and performance of the MOF thin films [163, 164]. The LbL deposition technique can be combined with other thin film deposition methods, thus allowing to integrate f‐MOF thin films in more complex systems, enabling the development of composite materials and advanced devices such as light‐emitting displays [165].

Hetero‐multilayer thin films can be efficiently fabricated via LbL assembly on functionalized substrates. Following the LbL assembly of an initial surface‐anchored MOF (SURMOF) thin film, the exposed uncoordinated functional groups at the surface of the thin films serve as anchoring sites for the subsequent growth of a secondary MOF layer, enabling the facile construction of heterostructured architectures with distinct properties [166]. The ability to seamlessly combine these films with other functional layers opens up new possibilities for the design of multifunctional materials and optoelectronic applications. In Ln‐MOFs, the shielded 4f orbitals of trivalent lanthanide ions typically do not engage in coordination bonding, often leading to isostructural frameworks across different lanthanides when using the same organic MOF linker. The LbL assembly emerges as a highly suitable approach for constructing hetero‐multilayer f‐MOF thin films, as it offers significant advantages over mixed‐metal MOF thin films, particularly in terms of precise control over emission properties and reduced unwanted energy transfer. By employing heteroepitaxial architectures, bilayer systems enable tunable luminescence with predictable color output, as demonstrated in Ln‐SURMOFs, where the separation of Tb(III) and Eu(III) layers minimizes direct energy transfer, unlike mixed‐metal bulk MOFs where random ion distribution leads to inefficient emission tuning. Additionally, multilayer configurations enhance thermal sensitivity in optical thermometers by strategically isolating lanthanide layers, thereby improving the accuracy of ratiometric temperature measurements compared to mixed systems. These advantages make hetero‐multilayer f‐MOFs highly suitable for advanced optoelectronic applications, including white‐light emission devices and sensitive optical sensors [19, 167, 168].

As summarized in Table 5, the synthesis of f‐MOFs often requires precise control over reaction conditions, making pump‐assisted and dipping LbL methods particularly advantageous for the fabrication high, optical quality thin films [168, 169, 170]. In a seminal study by Yang and coworkers (2017), europium‐based MOFs (Eu‐NDC) were deposited onto hydrolyzed polyacrylonitrile (HPAN) substrates via LbL growth, yielding thin films with a high density of accessible open pores [171]. This structural feature significantly enhanced the material's efficacy as a sensor for aqueous formaldehyde detection. Further developments by Chen et al. demonstrated the heteroepitaxial growth of Tb/Eu‐MOF thin films with controlled thickness and preferred crystallographic orientation, while also minimizing energy transfer between lanthanide ions‐enabling precise tuning of emission properties [168]. Building upon these advances, triple‐layer Tb/Eu/Gd‐MOF architectures were subsequently engineered for solid‐state white‐light‐emitting applications [172]. More recently, Chen et al. (2023) reported the LbL epitaxial synthesis of luminescent, transparent MIL‐103 MOF thin films exhibiting exceptional sensitivity as optical thermometers [167]. The same research group also achieved the LbL assembly of high quality crystalline uranium‐based MOF thin films, as shown in Figure 6f–h, wherein uranyl units adopted a highly symmetrical coordination geometry within a two‐dimensional framework, resulting in two distinct thermally activated emission bands [170]. Further details regarding the application of this uranium‐based MOF thin films is presented in Section 4.2.

The exceptional controllability of the LbL growth process further extends to the encapsulation of guest molecules during film deposition, significantly broadening the versatility of this self‐assembly technique. In 2012, Wöll, Wickleder and colleagues loaded europium b‐diketonate complexes into HKUST‐1 SURMOFs, as illustrated in Figure 6i, resulting in enhanced photonic absorption efficiency [173]. In 2015, Gu et al. demonstrated the incorporation of lanthanide coordination compounds into HKUST‐1 thin films via LbL assembly, establishing a highly precise and efficient approach for fabricating high‐performance white light‐emitting devices [174]. Building upon this work, the same group subsequently achieved the encapsulation of achiral lanthanide complexes within chiral MOF thin films using LbL assembly. As shown in Figure 6j, the resulting heterostructures exhibited pronounced circularly polarized luminescence (CPL) with a high dissymmetry factor (g), highlighting the potential of LbL techniques for engineering advanced photonic materials [175].

**TABLE 5:** Comparison of f‐MOF thin films by LbL assemble.

LbL approach	Substrate	Metal	Organic ligands	Self‐supporting	Application	Operational medium	Literature
Dipping method	Au wafer with SAM	Eu, Cu	BTC	N	Eu compound loading and Photonic Antennae	Air	[173]
Dipping method	HPAN film	Eu	NDC	Y	Fluorescence sensors for formaldehyde	Aqueous (Sensing)	[171]
Dipping method	FTO glass	Ce(III)	TCPP	N	Highly Efficient Oxygen Evolution Reaction	Aqueous (Catalysis)	[176]
Pump method	Quartz glass/Au wafer	Eu,Tb	BTC	N	Energy transfer control	Air	[168]
Pump method	Quartz glass	Eu, Tb, Gd	BTC	N	White‐Light Emission	Air	[172]
Pump method	Quartz glass /Au wafer	Eu,Tb, Gd,Cu	PDC; BTC	N	White‐Light Emission	Air	[174]
Pump method	Quartz glass	Eu,Tb	tFBDC; tClBDC; tBrBDC	N	Halogenated ligands control “antenna effect”	Air	[169]
Pump method	Quartz glass	Eu, Tb, Gd	BTB	N	Fluorescent thermometer	Air (Sensing)	[167]
Pump method	Quartz glass	Eu,Tb, Gd, Zn	Dcam; Lcam; dabco	N	Tunable chiroptical application	Air	[175]
Pump method	Quartz glass	U(VI)	BTC	N	Fluorescent thermometer	Air (Sensing)	[170]
Spray method	Si and Al substrates	Gd	BTC	N	Converter Layer for neutron detector	Air (Radiation)	[177]

### 2.3 MOF Particles Deposition/In Situ Solvothermal Synthesis

**FIGURE 7:**
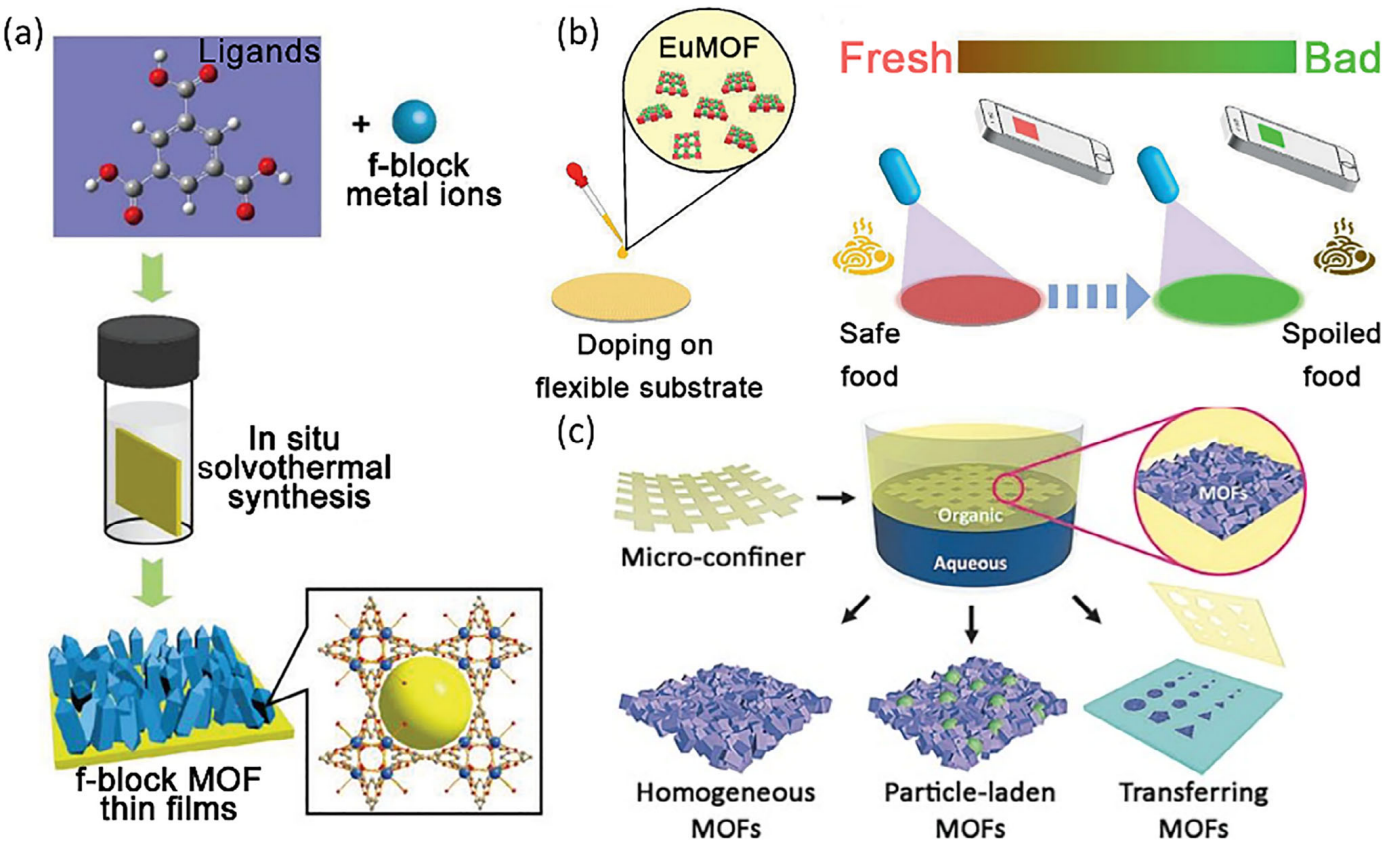
(a) Schematic diagram for the preparation of f‐MOF thin films by in situ solvothermal synthesis; Reproduced with permission [183]. Copyright 2019, Wiley‐VCH; (b) Schematic diagram of the EuMOF‐FITC composite film by MOF particles deposition method for visual monitoring of food freshness; Reproduced with permission [181]. Copyright 2021, Wiley‐VCH; (c) Schematic illustration of micro‐confined interfacial synthesis capable of delivering MOF superstructures in homogeneous single phases (HKUST‐1, ZIF‐8, LnBTC), heterogeneous phases (particle‐laden MOF), and subsequent transfer of the free‐standing MOF patterns on various substrates. Reproduced with permission [179]. Copyright 2016, Wiley‐VCH.

Early f‐MOF materials were primarily synthesized as crystalline or powder‐based particles through hydrothermal or solvothermal methods. Consequently, one of the earliest approaches for fabricating f‐MOF thin films involved either the direct deposition of MOF particles onto a substrate or the in situ hydrothermal/solvothermal synthesis of thin films by immersing the substrate under the same synthesis conditions, as exhibited in Figure 7. In 2010, Qiu, Lercher, and Zhang reported the first f‐MOF films by depositing MOF particles onto a substrate using the spin‐coating method [178]. As summarized in Table 6, significant advancements in f‐MOF film fabrication have been realized through various innovative strategies. The primary research impetus stems from the critical role of film formation as a pivotal step in translating advanced f‐MOF materials into functional devices. For instance, MOF superstructures have been obtained by interfacial MOF synthesis within micro‐confined environments, enabling precise control over film morphology and assembly [179]. Additionally, secondary in situ growth of Eu‐BDC on UiO‐66 has been demonstrated, achieving epitaxial growth with the same ligand, thus enhancing structural integration [180]. Post‐synthetic modification of Eu‐MOF with fluorescein 5‐isothiocyanate (5‐FITC) has been utilized to develop real‐time visual food freshness monitoring sensors, showcasing the potential of functionalized Ln‐MOF thin films in food safety applications [181].

**TABLE 6:** MOF particles deposition/In situ solvothermal synthesis.

Metal	Organic ligands	Film preparation method	Self‐supporting	Application	Operational medium	Literature
U(IV), Th(IV)	H_2_TNDA	Deposition of Th&U‐MOF on FET device	N	Semiconducting layer in two‐LED fail‐safe circuit	Air (conducting)	[184]
U(VI)	TPE	Deposition of An‐MOFs on LEDs	N	White light emission	Air	[185]
Dy, Eu, Tb	BTC	Spin‐coating deposition of Ln‐MOF nanoparticles on substrate	N	Ln‐MOF film preparation method	N/A	[178]
In,Eu, Tb,Dy,Sm	BTC	In situ solvothermal synthesis of Ln‐MOFs on supports with platinum nanoparticles	N	Fluorescence sensors for volatile thiols	Vapor (Sensing)	[186]
Eu	Oba	Filtration method using microcrystals	N	X‐ray imaging	Air & Aqueous	[187]
Eu	BQDC	Solvothermal deposition of Ln‐MOFs on ITO substrate	N	Fluorescence sensors for Hg^2+^	Organic solvent (Sensing)	[188]
Eu, Tb, Al	Trimellitic acid	Ln ions@Al‐MOFs films prepared by chemical solution deposition	N	White Light emission	Air	[189]
Eu,Tb, Yb,Al	TTA, TAA	Deposition of MOF particles on Ln^3+^ coated substrate	N	Ln‐containing MOF film preparation method	N/A	[190]
Eu,Tb, Al	BPYDC	Ln ions@Al‐MOFs films prepared by spin coating method	N	Anti‐counterfeiting	Air	[191]
Eu, Tb, Zr	BPYDC	Deposition of Ln ions@UiO‐MOFs on UV‐LED chip	N	White light emission; Fluorescent thermometer	Air	[192]
Eu, Tb, In	BTC	In situ solvothermal synthesis of Ln^3+^ ions@MIL‐100	N	Anti‐counterfeiting	Air	[193]
Eu, Tb, Ce(III)	BTC	Micro‐Confined Interfacial Synthesis	Y	Contact‐transferred method	N/A	[179]
Tb, Zn	BDC; BTC; 2,6‐NDC	In situ growth of Ln ions@MOF‐5 on ZnO micronanoarrays	N	Fluorescence sensors for small molecule	Vapor (Sensing)	[194]
Eu	BQDC	Ln‐MOFs grow on Co_3_O_4_ nanowire arrays of stainless‐steelwire mesh	N	Fluorescence sensors for Nitrofuran antibiotics	Aqueous (Sensing)	[195]
Eu, In	1,2,4‐BTC	Ln ions@MIL‐124 grow on porous Al_2_O_3_	N	Fluorescence sensors for ammonia	Vapor (Sensing)	[196]
Eu	Dichlorophthalic acid	Ln‐MOFs dispersed in glue and coated on substrate	N	Fluorescence sensors for Cr_2_O_7_^2–^	Aqueous (Sensing)	[197]
Eu,Tb	BTC	Dip‐coating deposition of Ln‐MOF monodisperse crystals on substrate	N	Fluorescence sensors for pharmaceuticals	Aqueous (Sensing)	[198]
Eu(III), Zr(IV)	NH_2_‐BDC	In situ secondary growth Ln‐MOFs on UiO‐MOF films	N	Fluorescence sensors for sulfur dioxide	N_2_ gas (Sensing)	[180]
Eu,Tb	BTC	Solvothermal synthesis grow on substrate	N	Fluorescent thermometer	Air (Sensing)	[183]
Tb	BTC	Ultrasonic spray deposition of Ln‐MOFs on substrate	N	Ln‐MOF film preparation method	N/A	[199]
Eu,Tb	2,6‐PDC	Deposition of Ln‐MOFs@Laponite on substrate	N	Fluorescence sensors for metal ions	Aqueous (Sensing)	[200]
Eu	BTEC	Deposition of Ln‐MOFs on oxidized cellulose nanofibrils	Y	Fluorescence sensors for copper ions	Aqueous (Sensing)	[201]
Tb	Salicylic acid	Deposition of Ln‐MOFs on test strip	N	Fluorescence sensors for tetracycline antibiotics	Aqueous (Sensing)	[202]
Ce(IV), Zr(IV)	BDC	Deposition of Ce‐UiO‐MOF particles on porous diatomite layer	N	Treatment of oily wastewater containing polyacrylamide	Wastewater (Separation)	[203]
Eu, Tb, Er, Nd, Zr	BPYDC	Deposition of Ln compounds@UiO‐type MOFs on substrate	N	Fluorescence sensors for ammonia	Air (Sensing)	[204]
Gd	Formylbenzoic acid	Deposition of Ln‐MOFs on conductive layer	N	Tunable Light Emitting Diodes	Air	[205]
Eu	NH_2_‐BDC; Phen	Ln‐MOF particles dropwise dispersed onto glass fiber films	N	Fluorescence sensors for biogenic amine; food monitoring	Vapor (Sensing)	[181]
Eu, Gd, Tb, Dy	Hpbmc	In situ self‐assembled Ln‐MOFs on PAN/PEM membrane	N	Fluorescence sensors for MnO^4−^ and ascorbic acid	Aqueous (Sensing)	[206]
Eu, Tb, Gd	BTC	Deposition of Ln‐MOFs@colloidal solution on substrate	N	Luminescent film preparation method	N/A	[207]
Yb, Tm, Zn	Methylimidazole	UCNP/DCM@ZIF‐8 in one‐pot solid‐confinement conversion process	N	Upconversion white light Emission	Air	[208]
Eu, Tb	TATB	Deposition of Ln‐MOF particles and attapulgite clay onto test paper	N	Fluorescence sensors for 2,6‐dipicolinic acid &Cu^2+^	Aqueous (Sensing)	[209]
Eu,Tb	BTC	Deposition of CH_3_NH_3_PbBr_2_@Ln‐MOF nanoparticals on ITO glass	N	Switchable memory	Air (photoelectricity)	[210]
Ce(IV)	BTC	Ce‐MOF on polypropylene separator by in‐situ growth	N	Inhibits the shuttle effect in lithium‐sulfur batteries	Solid (batteries)	[211]
Tb	BTC	Deposition of Ln‐MOFs on ZnO nanowires	N	Alternating current electroluminescence emissive layer	Air (electroluminescence)	[182]
Eu	BTC	Deposition of Ln‐MOF on conductive layer	N	UVC Photodetector for Invisible Fire	Air (Sensing)	[212]
Eu	DHTA	Ln‐MOFs coat on TiO_2_ Silk Fabric	N	Fluorescent thermometer	Air (thermometer)	[213]
Tb, Zn	BDC	Tb(III)@MOF‐5/ZIF‐8 thin film by solvothermal method deposition	N	Two‐dimensional conducting photodetectors	Air (photodetector)	[214]
Tb	H_2_BTBC	Deposition of Ln‐MOFs on substrates	N	Fluorescence sensors for ascorbic acid	Organic solvent (Sensing)	[215]
Eu,Tb	BTA, H_3_ICA	Depositing Ln‐MOF particles onto filter paper	N	Fluorescence sensors for metal ions and pharmaceuticals	Aqueous (Sensing)	[216]
Eu	BTA	Deposition of carbon dots & Ln‐MOFs & carboxymethyl cellulose on LED chips	N	White Light emission	Air	[217]
Ce(III), Tb	Imidazolate‐anion	In situ deposition method of Ln‐MOF films	N	Organic melt, electride, and CVD induced in situ deposition	N/A	[218]
Ce(III), Ni(II), Cu(II)	BTC	Deposition of Ni–Ce–Cu MOF on electrode	N	Electrochemical sensor for L‐cysteine	Aqueous (Sensing)	[219]

Recent advancements include the deposition of Ln‐MOFs onto ZnO nanowires, forming an alternating current electroluminescent emissive layer, which represents a significant step toward MOF‐based light‐emitting devices [182]. These developments highlight the continuous evolution of f‐MOF thin film synthesis and their expanding applications in sensing, optoelectronics, and functional coatings. However, simple direct deposition approaches frequently result in island‐like growth (Volmer‐Weber mode), producing films with significantly more defects compared to mixed‐matrix membranes (MMMs). Additionally, films prepared by methods such as layer‐by‐layer (LbL) assembly present challenges in precise thickness control and exhibit inferior mechanical strength relative to electrospun membranes. As illustrated in Figure 1, these limitations have led to the gradual replacement of direct deposition techniques for f‐MOFs film fabrication by alternative methods offering superior performance characteristics in recent years.

### 2.4 Electrodeposition

**FIGURE 8:**
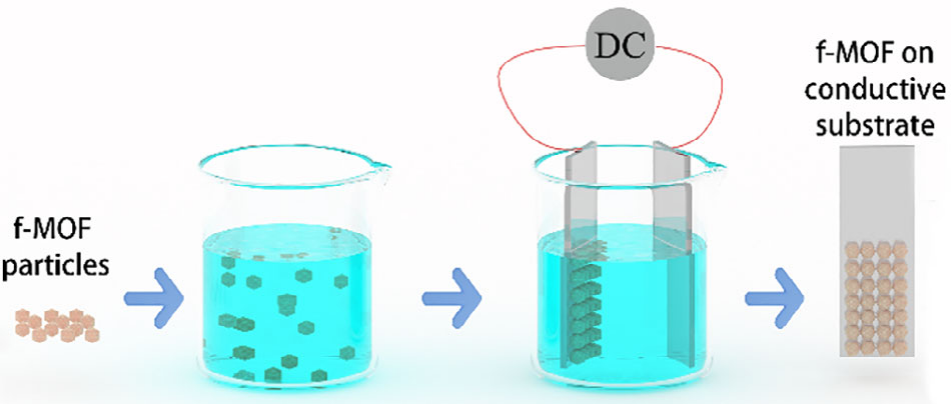
General scheme for electrodeposition of f‐MOFs onto conductive substrate.

Electrodeposition has emerged as a promising technique for the fabrication of Ln‐MOF thin films, offering a high‐throughput and scalable approach for large‐area thin film production [220, 221, 222]. This method employs an applied electrical potential to a conductive substrate submerged in an electrolyte solution containing lanthanide ions and organic linkers, inducing redox reactions at the substrate interface that promote the nucleation and growth of Ln‐MOF thin films.(Figure 8) The electrodeposition process enables precise control over film morphology and thickness through modulation of key parameters, including applied voltage, current density, and deposition time, yielding highly uniform and adherent films. However, as evidenced in Table 7, significant variations exist in deposition mechanisms across different studies, particularly regarding electrode positioning and required voltage thresholds during the deposition process. Notably, this technique stands out for its simplicity, scalability, and compatibility with complex substrate geometries, making it particularly advantageous for the development of advanced Ln‐MOF thin films for diverse applications.

**TABLE 7:** Comparison of f‐MOF thin films by electrodeposition method.

Substrate	Electrode cell	Deposition electrode	Current Or voltage	Metal	Organic ligands	Self‐supporting	Application	Operational medium	Literature
Zinc plate	Three‐electrode cell	Cathode /working electrode	5 mA/cm^2^	Eu(III)	BTC	N	Fluorescence sensors for explosives	Organic solvent (Sensing)	[227]
FTO glass	Two‐electrode cell	Cathode	0.4 mA/cm^2^	Eu(III)	H4BPTC	N	Fluorescence sensors for carbonate	Aqueous (Sensing)	[220]
FTO glass	Two‐electrode cell	Cathode	0.6 mA/cm^2^	Eu(III), Gd(III) Tb(III)	Hemimellitic acid	N	Tunable White‐Light and Multicolor Emission	Air	[228]
FTO glass	Three‐electrode cell	Cathode /working electrode	0.4 mA/cm^2^	Tb(III)	Succinate	N	Fluorescence sensors for Cu^2+^	Aqueous (Sensing)	[221]
FTO glass	Two‐electrode cell	Cathode	0.5 mA/cm^2^	Eu(III)	2,6‐NDC	N	Fluorescence sensors for picric acid	Aqueous (Sensing)	[229]
Zinc Plate/FTO/ITO glass	Two‐electrode cell	Positive electrode	N/A	Eu(III), Tb(III)	BTC	N	Fluorescence sensors for TNT and Ethanol	Aqueous and gas (Sensing)	[223]
FTO glass	Two‐electrode cell	Positive electrode	N/A	Eu(III), Tb(III), Zr(IV)	BTA	N	Fluorescent thermometer	Air (Sensing)	[224]
FTO with polystyrene arrays	Two‐electrode cell	Cathode	−0.4 mA/cm^2^	Eu(III), Tb(III)	H_2_TDA	N	Fluorescence sensors for organic amines	Vapor (Sensing)	[225]
Porous anodic oxide	Two‐electrode cell	Cathode	20 V	Ce(III)	BTC	N	Improve the corrosion resistance of aluminum alloy	Salt Vapor (corrosion)	[230]
Porous anodic oxide	Two‐electrode cell	Cathode	12 V	Ce(III)	BTC	N	Metal corrosion protection	Salt Vapor (corrosion)	[231]
ITO‐glass	Two‐electrode cell	Cathode /working electrode	1.2 mA/cm^2^	Eu(III), Tb(III)	Fumaric acid, BDC, BPDC	N	X‐ray imaging	Air (scintillating)	[226]

In 2014, Yang and coworkers presented Ln‐MOF thin films, fabricated by electrodeposition, exhibited precise control over film growth under mild conditions, enabling the formation of dense, uniform, and water‐stable films with enhanced luminescent properties, making them ideal for applications in sensing and environmental monitoring [220, 221, 222]. Cao and coworkers demonstrated the ability of electrodeposition for fabricating Ln‐MOF thin films to rapidly and facilely deposit continuous and dense films on unmodified, low‐cost substrates within minutes. The approach offers high efficiency and cost‐effectiveness. It also holds significant potential for practical applications in sensing, lighting‐emitting devices, and temperature detection [223, 224]. Wu and coworkers demonstrated this method offers enhanced optical properties, including structural and fluorescent colors, and the ability to create dual‐module sensors for efficient multi‐analyte detection, making it a versatile platform for environmental monitoring and sensing applications [225].

In recently, Mohammed and coworkers also presented an in situ electrochemical deposition method for fabricating compact Ln‐MOF thin films, which are tailored for high‐resolution X‐ray imaging [226]. The electrochemical approach enables precise control over the growth of defect‐free, continuous MOF thin films, significantly reducing light scattering and enhancing material density, thereby improving spatial resolution in X‐ray imaging. The key advantages of this method include the ability to produce uniform, dense films with minimal light scattering, leading to superior imaging performance compared to traditional methods, and the potential for scalable production of high‐performance scintillators for medical and security applications. To date, the electrochemical deposition of An‐MOF thin films remains unexplored in the literature. However, such an approach could offer a promising route for the rapid fabrication of ultrathin, uniform α‐particle‐emitting sources on functional substrates, which would hold significant potential for applications in nuclear energy and radiation detection.

## 3 Advanced Applications of Ln‐MOF Thin Films

**FIGURE 9:**
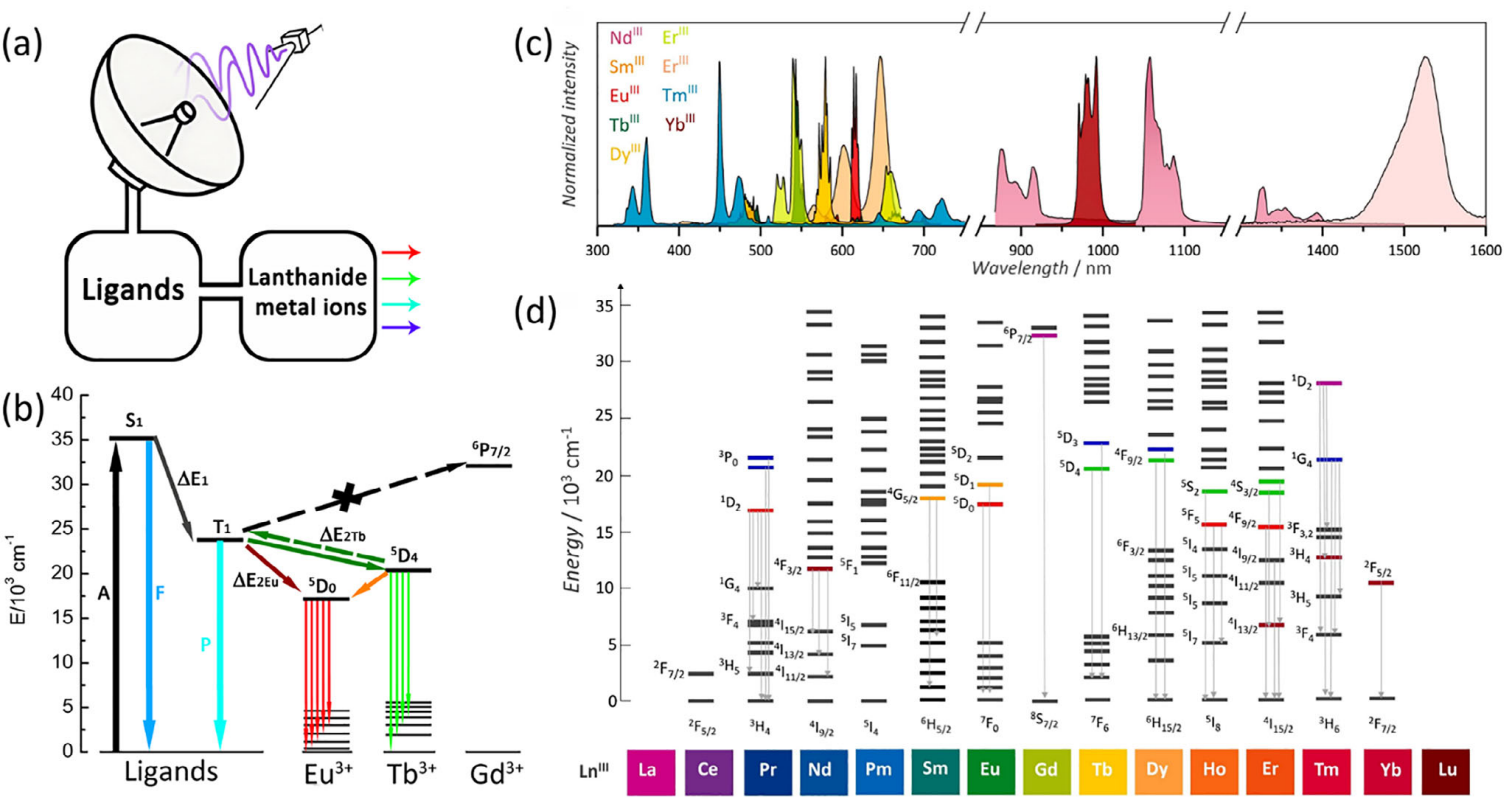
(a) Schematic representations of the “antenna effect”; (b) Schematic representation of the energy levels of organic ligands and Ln^3^^+^ ions (Eu^3^^+^, Tb^3^^+^, Gd^3^^+^), illustrating the typical “antenna effect” observed in Ln‐MOFs. (A = absorption; F = fluorescence; P = phosphorescence; S = singlet; and T = triplet; ΔE_1_ = energy gap between S_1_ and T_1_ of ligands; ΔE_2Eu_ = energy gap between T_1_ and excited state of Eu^3+^ ions; ΔE_2Tb_ = energy gap between T_1_ and excited state of Tb^3+^ ions). (c) Emission fingerprint of some LnIII highlighting the main emissions within the UV‐to‐IV spectral window. (d) Partial Dieke diagram representing the energy levels arising from the ^4^f_n_ configurations of Ln(III) and their main electronic transitions. Figure 9c,d are reproduced with permission [233]. Copyright 2025, Elsevier.

Lanthanide ions are well known for their sharp and stable emission bands [9]. However, because f–f transitions are Laporte‐forbidden, individual lanthanide ions have intrinsically low absorption cross‐sections, making them inefficient at directly capturing photons for electronic excitation. Fortunately, in lanthanide‐based coordination compounds such as Ln‐MOFs, organic ligands can serve as sensitizers by efficiently absorbing light and transferring the energy to the lanthanide ions, thereby enabling their excitation [232]. The excited electrons then transfer from characteristic resonance energy levels back to the ground state, leading to a significant enhancement in photoluminescence efficiency. The energy levels of all trivalent lanthanide ions and their characteristic emission bands are shown in Figure 9. This phenomenon, commonly referred to as the “antenna effect”, serves as a fundamental principle underlying the widespread application of Ln‐MOFs in optical and photonic technologies. Inheriting the nature of Ln‐MOFs, Ln‐MOF thin films have emerged as a versatile and highly functional class of materials, offering significant potential in various advanced technological applications due to their unique structural and optical properties. These materials combine the inherent advantages of lanthanide ions—such as sharp emission bands, long luminescent lifetimes, and high thermal stability—with the structural tunability of MOFs, resulting in films that are particularly suitable for integration into devices.

### 3.1 Sensing Application of Ln‐MOF Thin Films

One of the primary applications of Ln‐MOF thin films is in the field of sensing. The versatile ligand design and tunable pore structures of Ln‐MOFs allow their luminescent properties to be highly responsive to external stimuli or guest molecules, modulating key photophysical processes such as ligand‐to‐metal energy transfer, intersystem crossing, and non‐radiative decay rates. This tunability enables the selective detection of specific analytes through distinct fluorescence responses. Furthermore, in multi‐lanthanide‐doped Ln‐MOF systems, energy transfer (*η*_ET_) between different lanthanide ions can be described by the efficiency equation [234]:

$$\eta_{ET}=1-\frac{\tau_{DA}}{\tau_{D}}$$

where τ_*DA*_ and τ_*D*_ represent the emission lifetime of acceptor ions (e.g., Eu^3^^+^ in the Eu‐Tb system) and donor ions (e.g., Tb^3^^+^ in the Eu‐Tb system), respectively, in the in multi‐lanthanide‐doped Ln‐MOF systems.

The stimulus‐dependent variation in energy transfer rates among lanthanide ions provides an additional mechanism for the highly discriminative sensing of external stimuli, making multi‐lanthanide Ln‐MOFs particularly promising for advanced sensing applications.

**FIGURE 10:**
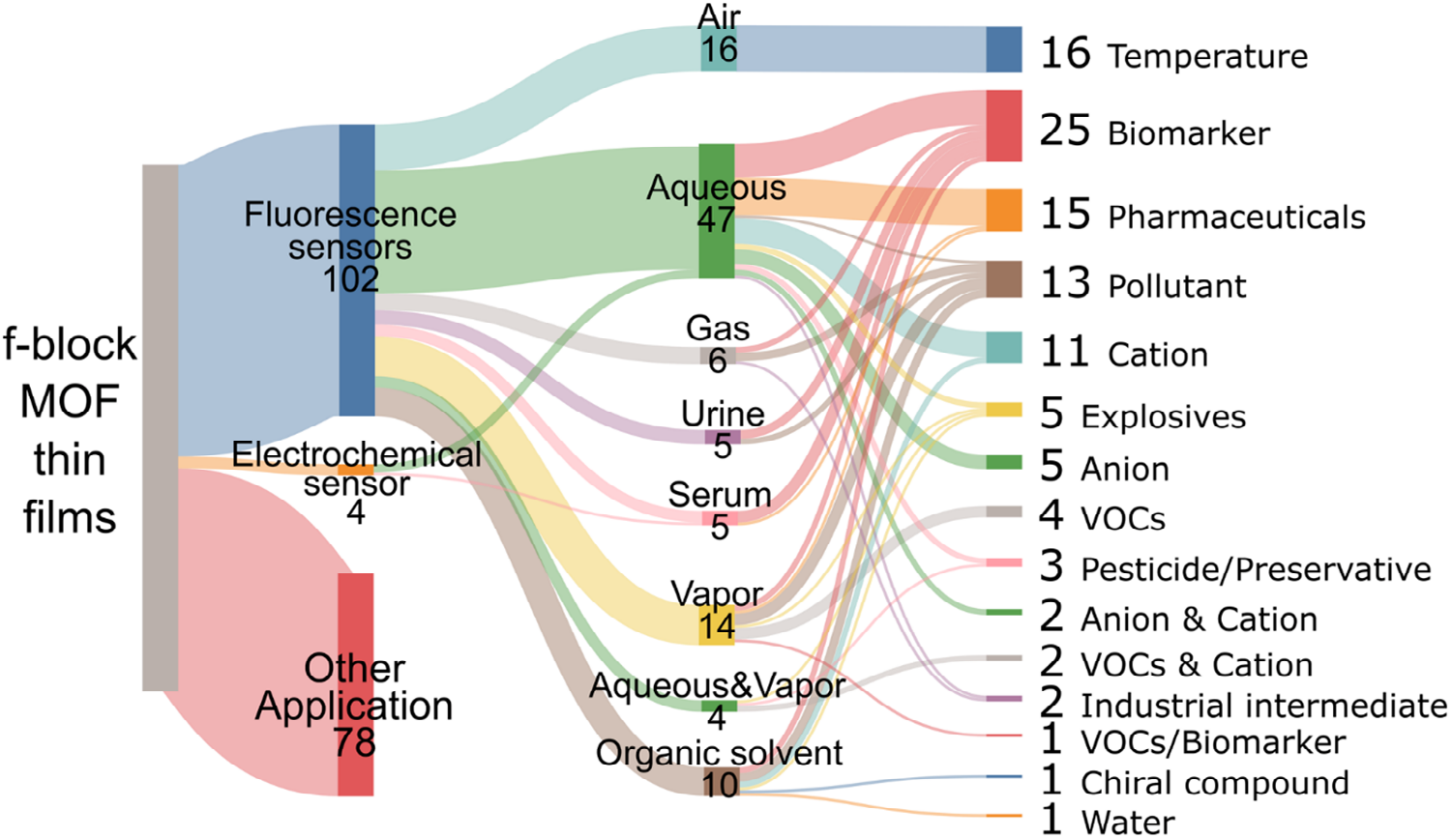
Sankey plot representing the correlation between operational media and target analytes in sensing applications of f‐MOF thin films.

As demonstrated in Figure 10, the luminescent properties of lanthanides make these films highly effective for optical sensing, enabling the detection of a wide range of analytes, including temperature, gas molecules, metal ions, and organic compounds. The high surface area and porosity of the thin films facilitate efficient interaction with target molecules, enhancing sensitivity and selectivity.

Fransaer and coworkers presented the flexibility of electrochemical synthesis for producing luminescent Tb‐MOF films on various substrates, which effectively detect explosives like 2,4‐dinitrotoluene (DNT) in both liquid and gas phases, underscoring their potential for security applications [227]. Wang et al. demonstrated that electrodeposited Terbium‐Succinate (Tb‐SA) thin films exhibit excellent water stability and a rapid, eye‐detectable response to Cu^2^^+^ ions, making them suitable for in‐field environmental monitoring [221].

Wang et al. highlight the advantage of Tb(III)‐functionalized CP coatings on ZnO micronanoarrays, which exhibit selective luminescence quenching for acetone detection, offering a low‐cost and convenient sensing platform with potential applications in volatile organic compound (VOC) monitoring [194]. The study by Gao et al. demonstrated that mixed‐crystal Ln‐MOF thin films enable self‐referencing and self‐calibrating luminescent sensing of pharmaceuticals by leveraging the distinct emission intensity ratios of Eu^3^^+^ and Tb^3^^+^ transitions, providing a stable and accurate method for molecular recognition and concentration quantification [198]. In the study of Cao and coworkers, the Ln@UiO‐66‐Hybrid thin films exhibit dual‐emitting properties with high relative sensitivity (up to 4.26% K^−^^1^) for ratiometric temperature sensing, leveraging the wide emission range and stable luminescence of lanthanide ions and organic ligands within the MOF structure [224].

In 2020, Chen and coworkers achieved a novel mixed‐lanthanide polyMOF membrane designed for ratiometric temperature sensing applications [30]. Using a photoinduced post‐synthetic copolymerization strategy, the researchers created a flexible, elastic, and processable membrane that combines the temperature sensing capabilities of Ln‐MOFs with enhanced mechanical properties. This membrane demonstrates robust stability under harsh conditions, such as high humidity and extreme pH levels (0‐14), enabling accurate temperature mapping in challenging environments. This innovation highlights the potential for practical applications of Ln‐MOF‐based luminescent thermometers across various fields, including biology, chemistry, and engineering.

In 2021, Yuan and coworkers discussed the development of a Ln‐MOF thin film for detecting antibiotic residues in food [235]. This bimetallic TbEu‐MOF thin film demonstrates high sensitivity and selectivity for sulfamerazine and malachite green, utilizing ratiometric fluorescence changes for detection. A smartphone application aids in visualizing results, making it a practical tool for ensuring food safety. Meanwhile, Ouyang and coworkers demonstrated enhanced water stability and detection sensitivity for UO_2_^2^^+^ (LOD of 0.75 nM) in PVTP⊂Tb‐TBT thin films, by integrating polymeric ligands as molecular scaffolds and antennae, enabling efficient energy transfer and selective analyte enrichment for on‐site environmental monitoring [124]. Cui and coworkers developed a chiral isostructural Ln‐MOF nanosheet MMMs were utilized as a fluorescence‐sensing platform, demonstrating high enantioselectivity in the detection of terpenes and terpenoids. The thin‐film configuration of these materials enhances recyclability and mitigates clogging issues, making them advantageous for practical sensing applications [36].

A dual‐lanthanide MOF thin film sensor for detecting trace water in organic solvents was introduced by Xiao and coworkers [236]. The dual‐lanthanide MOF utilizes a urea‐containing ligand and innovative response mechanisms to achieve high sensitivity and a broad detection range. The sensor changes its luminescence from red to green based on water content, functioning as a “traffic light” indicator. This design includes a logic device for simple, intelligent water detection, and the MOF‐loaded paper microsensor enables real‐time visual water assay with the help of a smartphone.

Liu and coworkers introduced an Ln‐MOF doped with Eu^3^^+^ and Tb^3^^+^ for highly sensitive and selective ethanol vapor detection [80]. The ethanol binds to the framework, changing the energy transfer efficiency between the ions to enable luminescence‐based detection. This film is portable, processable, and exhibits good repeatability. This membrane offers excellent mechanical properties and stability under extreme conditions, facilitating temperature mapping in harsh environments. While both utilize Ln‐MOFs' luminescent properties, the former is optimized for temperature sensing, and the latter for ethanol vapor sensing.

In summary, the sensing applications of Ln‐MOF thin films primarily arise from their stimuli‐responsive optical properties, which are modulated by interactions between the periodic coordination structure of Ln‐MOFs, and the target analytes (small molecules, temperature, etc.). Key mechanisms include charge transfer between ligands and metal centers, fluorescence resonance energy transfer (FRET), and dynamic quenching effects, all of which can alter the luminescence behavior of the thin films [237, 238]. These processes lead to detectable changes in emission intensity, wavelength shifts, or lifetime variations, enabling visible or spectroscopic responses. The structural tunability and high porosity of Ln‐MOFs further enhance their selectivity and sensitivity toward specific analytes, making them promising candidates for optical sensing platforms. The thin‐film morphology not only facilitates rapid analyte diffusion but also allows for integration into device‐based sensing systems, highlighting their practical potential in environmental monitoring, chemical detection, and biomedical diagnostics [19].

### 3.2 Light‐Emitting Diodes of Ln‐MOF Thin Films

Beyond sensing applications, Ln‐MOF thin films have emerged as promising candidates for light‐emitting diodes (LEDs). The tunable emission properties of lanthanides, enabled by the strategic selection of specific metal ions and organic linkers, facilitate the rational design of LEDs with tailored chromatic output and enhanced performance. Notably, the structural versatility of Ln‐MOFs allows for the incorporation of multiple lanthanide centers within a single framework, enabling multicolor emission through red‐green‐blue (RGB) mixing strategies. The resulting photoluminescence profiles can be precisely characterized using Commission Internationale de l'Éclairage (CIE) chromaticity coordinates, providing an accurate and systematic approach to color tuning and quantification. Such adaptability positions Ln‐MOF thin films as a highly versatile platform for advancing next‐generation optoelectronic devices, particularly in lighting and display technologies.

Recent studies have demonstrated the potential of Ln‐MOF thin films as efficient solid‐state emitters for LED applications. Zhou and Yan reported a strategy for imparting tunable and white‐light luminescence to Al‐MIL‐53‐COOH nanocrystals by encapsulating Eu^3^^+^ and Tb^3^^+^ ions, achieving dual‐emissive behavior through both ligand‐centered and lanthanide‐based emissions [189]. These films exhibited high quantum yields, good thermal stability, and compatibility with aqueous environments, indicating suitability for practical devices. Complementarily, Gu, Zhang and coworkers developed a liquid‐phase epitaxy (LPE) approach to incorporate lanthanide coordination compounds into HKUST‐1 thin films with high encapsulation efficiency [174]. By modulating the ratios of Eu^3^^+^, Tb^3^^+^, and Gd^3^^+^ complexes, they achieved homogeneous, oriented films with tunable emission, including high‐quality white light, suitable for RGB‐based LED devices. Together, these studies highlight the structural control, emission tunability, and processability of Ln‐MOF thin films, positioning them as promising materials for next‐generation LED technologies.

In 2020, Chen et al. investigated the application of Ln‐MOF thin films in LED technology [172]. The study focuses on SURMOF devices that utilize heteroepitaxial architectures to achieve white‐light emission. These devices demonstrate efficient and stable luminescent performance, with the added capability of temperature‐dependent luminescence, which is valuable for creating smart lighting systems. The white‐light emission is achieved through a combination of lanthanide ions, providing high color purity and stability, which are critical for advanced LED applications.

**FIGURE 11:**
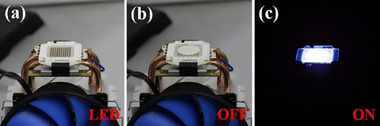
(a) The photograph of a 980 nm LED chip. Photographs of a rough white light‐emitting LED assembled from the upconversion nanoparticles (NaYF_4_:Yb,Tm@NaYF_4_) @ZIF‐8 thin film with a near‐infrared 980 nm LED chip when the LED is turned off (b) and turned on (c). Reproduced with permission [208]. Copyright 2021, Elsevier.

Deng and Peng employed Ln‐MOF thin films for LED applications due to their unique advantages in achieving high‐performance white‐light emission under near‐infrared (NIR) excitation [208]. The integration of lanthanide upconversion nanoparticles and organic dyes within a ZIF‐8 framework enables efficient energy transfer processes, resulting in a single‐phase phosphor with exceptional color quality (CIE coordinates of 0.33, 0.33), high color rendering index (CRI = 93), and tunable emission. The MOF matrix offers superior thermal and chemical stability, preventing aggregation‐induced quenching and ensuring long‐term durability, even under harsh conditions. Additionally, as shown in Figure 11, the thin‐film format simplifies device fabrication, allowing direct assembly onto commercial NIR LED chips. This approach overcomes limitations of traditional phosphors, such as UV toxicity and mismatched degradation rates, while leveraging the porous structure to isolate and stabilize luminescent components.

In 2022, Zhu and Wang explored the use of Ln‐MOF thin films in LED applications [239]. These nanocomposites exhibit dual‐mode luminescence and are used to enhance the efficiency and stability of white LEDs. By incorporating lanthanide ions into the MOF structure, the resulting thin films provide high color purity and stability, making them suitable for advanced lighting and display technologies. This study highlights the potential of Ln‐MOF thin films in improving LED performance and broadening their application in various fields.

### 3.3 Anti‐Counterfeiting Application

Effective anti‐counterfeiting materials must meet several key requirements: [240, 241, 242]. (1) high security, ensuring features are difficult to replicate; (2) multifunctionality, incorporating multiple authentication modes (e.g., optical, chemical, or mechanical responses); (3) durability, maintaining stability under environmental stressors; and (4) scalability, enabling cost‐effective mass production. Additionally, advanced anti‐counterfeiting technologies should integrate dynamic responsiveness (e.g., to light, heat, or chemicals) and machine‐readable features for enhanced verification.

Ln‐MOF thin films are particularly advantageous in this field due to their tunable luminescence, allowing precise control over emission colors and lifetimes via lanthanide ion selection and organic linker modification. Their high quantum yields and sharp emission bands enable distinct, easily identifiable patterns that resist duplication. Furthermore, Ln‐MOFs exhibit stimuli‐responsive behavior (e.g., thermochromism or photochromism), permitting dynamic security features. Their nanoscale porosity facilitates the incorporation of additional encryption elements, such as guest molecules or quantum dots, enhancing complexity. Compared to conventional materials (e.g., dyes or quantum dots), Ln‐MOF films offer superior chemical and thermal stability, ensuring long‐term functionality. Thus, Ln‐MOF thin films represent a next‐generation solution, combining high security, versatility, and practical feasibility for advanced anti‐counterfeiting applications.

In 2015, the study by Júnior and coworkers demonstrated the inkjet printing of Ln‐MOFs onto flexible substrates, such as plastic and paper, for anti‐counterfeiting applications [156]. The Ln‐MOF inks exhibited sharp, tunable emission under UV light, enabling the creation of covert, high‐security patterns. The method leverages rapid crystallization, adhesion stability, and resistance to mechanical/thermal stress, making it suitable for document authentication and product labeling. Subsequently, the dual‐switchable luminescent and chromic properties were demonstrated, which enable dynamic, stimuli‐responsive color and emission changes under external triggers such as light, temperature, or humidity [31, 56].

The nature of Ln‐MOFs, including high quantum yields, tunable emission colors, and ability to integrate with polymers like PDMS or PMMA enhance durability and flexibility, making them suitable for rewritable information storage and advanced encryption [83]. The study by Yu et al. (2024) introduces a water‐stable 2D lanthanide metal–organic framework (NIIC‐2‐Tb) with exceptional luminescence properties, achieving ultra‐sensitive detection of ofloxacin (OFX) at concentrations as low as 1.1 × 10‐9 M with rapid response times (6 s).144 Additionally, the unique photophysical properties of Ln‐MOFs, including time‐resolved luminescence and multi‐stimuli responsiveness, provide high‐security features that are difficult to replicate, further solidifying their role in next‐generation anti‐counterfeiting technologies [55, 161, 240].

### 3.4 Applications of Chiral Ln‐MOF Thin Films

Chiral MOFs are a class of porous crystalline materials with inherent chirality, offering precise control over enantioselective processes in catalysis, separation, and sensing, making them crucial for advancing pharmaceutical, chemical, and materials science applications [34]. Chiral Ln‐MOFs have emerged as a significant subclass of chiral MOFs due to their unique luminescent and magnetic properties, which are derived from the f‐electrons of lanthanide ions. These materials are constructed using enantiopure organic ligands or through spontaneous resolution, resulting in frameworks with well‐defined chiral environments. Ln‐MOFs exhibit exceptional potential in enantioselective applications such as catalysis [243], sensing [244], and separation [245], leveraging their high stability and tunable porosity. Their ability to combine chirality with luminescence also makes them promising candidates for circularly polarized luminescence (CPL) and nonlinear optics (NLO) [246, 247, 248]. The development of chiral Ln‐MOFs has expanded the toolbox for designing multifunctional materials with tailored properties for advanced technological and biomedical applications.

Building on these foundational developments, the fabrication of chiral Ln‐MOF thin films represents a critical step toward practical applications. Qiu and Ben presented the synthesis of chirality‐enriched MOFs (CE‐MOFs) using achiral building blocks and recoverable chiral dopants, which exhibits enantioselective recognition and separation capabilities [34]. The study demonstrated that CE‐MOFs, when incorporated into MMMs, enable chiral separation with performance influenced by solvent polarity, achieving an enantiomeric excess (ee) value of 9% in nonpolar solvents. The findings highlight the potential of CE‐MOFs for cost‐effective and scalable applications in chiral separation technologies. The creation of circularly polarized luminescence (CPL) thin films by incorporating achiral lanthanide complexes into chiral MOFs (chirMOFs) was described by Gu and coworkers [175]. Using a layer‐by‐layer encapsulation strategy, these films exhibit tunable chiroptical properties due to the unique combination of chiral porous MOFs and adjustable luminescent complexes. This enables the development of advanced materials for optical devices and sensors with applications in enantioselective detection and asymmetric catalysis.

### 3.5 Applications in Catalysis

The redox behavior of cerium differs markedly from other lanthanides due to its relatively low 4f ionization potential barrier, whereas most lanthanide ions exhibit significantly higher 4f ionization energies [249]. Consequently, unlike the majority of lanthanides, which lack accessible variable oxidation states, Ce stands out as the only lanthanide in Ln‐MOFs capable of efficient catalytic activity. This unique property arises from the reversible redox transition between Ce(III) ([Xe]4f^1^) and Ce(IV) ([Xe]4f^0^), which facilitates the generation of catalytically active sites essential for various chemical transformations [250]. Duan and coworkers demonstrated the potential of Ce‐MOF particles in asymmetric catalysis, where homochiral Ce‐MOFs exhibited excellent enantioselectivity in cyanosilylation reactions [243]. Truhlar and coworkers demonstrated the crucial role of Ce(IV) low‐lying 4f orbitals in promoting efficient ligand‐to‐metal charge transfer, which enhances charge separation and photocatalytic activity for applications like water splitting and CO_2_ reduction in Ce‐UiO‐66 particles [251].

However, due to the influence of current thin‐film fabrication processes on the catalytic sites of materials, reports on the preparation of Ce‐MOFs into thin films for catalytic applications remain quite limited at this stage. An electrospun nano‐Ce‐MOF (BIT‐58) thin films were reported by Wang and coworkers and utilized into catalytic application [131]. The thin film enhanced the catalytic performance by exposing more active sites (10× acid sites, 7× mesopore volume) due to reduced particle size (∼30 nm). With high MOF loading (70 wt.%), these films exhibit excellent mechanical stability, retained porosity (409 m^2^ g^−^^1^), and efficient mass transport. They achieve full conversion in Knoevenagel condensation and remain reusable. The method allows scalable fabrication and direct substrate coating, enabling inert surfaces to function as robust catalysts, ideal for continuous‐flow or batch reactions. Inspired from this work, we believe that combining Ce‐MOFs with exceptional catalytic activity and advanced thin‐film fabrication techniques (such as LbL growth or electrodeposition) could further expose additional catalytic active sites, ultimately yielding catalytic thin films with industrial potential. In addition, the stable tetravalent state of Ce has been frequently incorporated into UiO‐type MOFs structures [252]. Exploiting the high stability of Ce‐UiO‐type MOFs, significant progress has been made in adsorption and separation applications [138, 203].

The Ce(IV) ions in Ce‐UiO‐type MOFs, compared to Zr(IV) ions, exhibited lower proton affinity and higher proton transfer capability which facilitates the construction of hydrogen‐bonded networks and enhances proton conductivity in the composite membrane [48]. The Ce‐UiO‐type MOFs also exhibited strong antibacterial properties motivated by its intrinsic haloperoxidase‐like activity in the research of Wang and Li [138]. The electrospun Ce‐UiO‐MOF membrane provided a high specific surface area and enhanced adsorption capacity for the removal of 2,4‐dichlorophenoxyacetic acid from water.

In summary, Ln‐MOF thin films represent a burgeoning field of research with diverse applications in sensors, LED materials, anti‐counterfeiting, chiral applications, and catalysis application. Their unique combination of structural and physical properties, coupled with their tunability and integration potential, positions them as key materials for future advancements in these areas.

## 4 Advanced Applications of An‐MOF Thin Films

Although far less studied than Ln‐MOFs, An‐MOF thin films are attracting growing interest due to their distinctive structural and functional properties, positioning them promising for applications in radiation and thermal management. The broader range of accessible oxidation states in actinide ions, compared to lanthanides, stems from the weaker shielding of their 5f orbitals. A previous comprehensive review, from Schmidt and co‐workers, detailed the synthesis, structural design, and structure‐property relationships of An‐MOFs [18]. In a separate review, Shustova and co‐workers summarized subsequent developments, emphasizing applications in gas storage and separation, photophysics, catalysis, and electronics [29]. The group's subsequent work provided a comparative analysis of the characteristics and interrelationships of thorium‐, uranium‐, and zirconium‐MOF materials [253]. In contrast to these prior works on bulk materials, the scope of this section is specifically dedicated to the emerging research on An‐MOF thin films. Our discussion will evaluate the current progress in fabrication techniques and identify promising An‐MOF compositions with inherent properties amenable to processing as thin films. These distinctive electronic and bonding characteristics endow An‐MOFs with unique redox activity, harder Lewis acidity, and enhanced coordination flexibility. While isomorphism is frequently observed in Ln‐MOFs due to their similar ionic radii and oxidation states, it is more constrained in An‐MOFs as a result of greater variability in oxidation states and ionic radii. However, isomorphism remains feasible within homovalent actinide series. By incorporating high‐atomic‐number elements such as uranium and thorium into the modular MOF architecture, An‐MOF thin films synergize the intrinsic properties of actinides—including radioactivity and heavy‐element effects—with the structural tailorability and porosity of MOFs, further expanding their functional utility.

### 4.1 X‐Ray Scintillation and Radiation Protection

In the field of X‐ray detection and radiation protection, actinide elements, characterized by their high atomic number (Z), exhibit exceptional radiation absorption capabilities. Consequently, actinide‐element compounds incorporating high‐Z elements have emerged as promising materials for applications such as X‐ray scintillators and radiation shielding [254]. By selecting suitable ligands and actinide metal centers, An‐MOFs with unique structural features can be assembled, making them critical components in medical imaging, security scanning, and high‐energy physics experiments. Within this application domain, the fabrication of An‐MOF thin films represents a key research direction for advancing X‐ray scintillation and radiation protection technologies. In thin‐film form, An‐MOFs can simultaneously achieve rapid radiation response and comprehensive shielding performance. Moreover, the processability of thin films offers a versatile pathway for integrating An‐MOFs with other materials and interfaces, facilitating the development of composite systems for enhanced functional performance.

**FIGURE 12:**
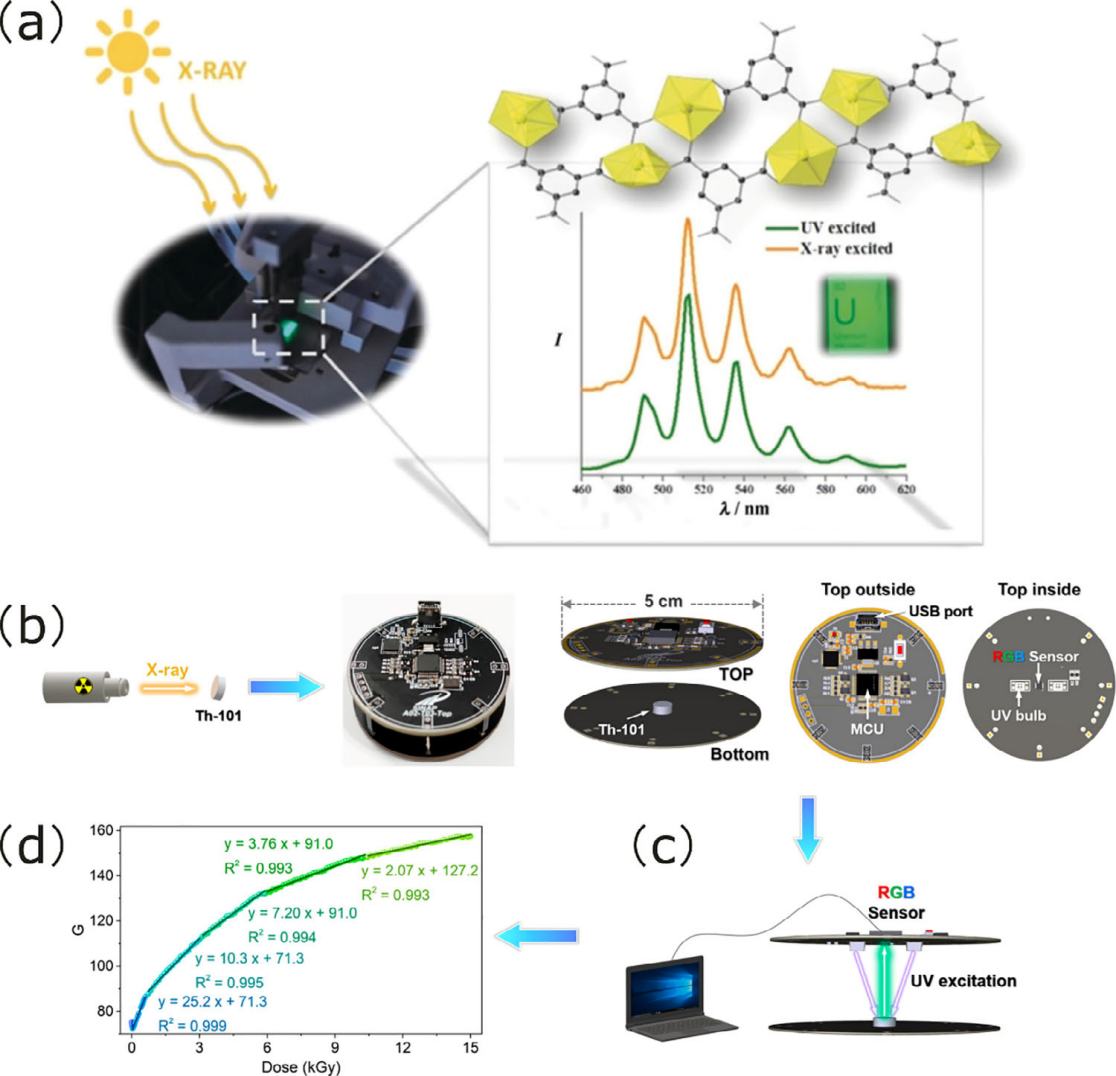
(a) Bright green emission of uranyl–organic framework (SCU‐9) excited by X‐ray in a powder X‐ray diffractometer. Reproduced with permission [255]. Copyright 2018, Wiley‐VCH; (b) Schematic illustrations and digital image of the lab‐built optoelectronic device integrating Th‐101 as the RGB‐based dosimeter. (c) Schematic illustration showing the design principle of the optoelectronic device for X‐ray dosimetry. (d) RGB integer (green) of Th‐101 as versus X‐ray dose. Reproduced with permission [256]. Copyright 2022, American Chemical Society.

Wang and coauthors explored the development of An‐MOF thin films for X‐ray scintillation applications [255, 257]. As illustrated in Figure 12, these uranyl‐based MOFs exhibit strong luminescent responses when exposed to X‐rays, making them effective scintillators for detecting ionizing radiation. The incorporation of actinide elements enhances both stopping power and light yield due to their high‐Z nature and unique charge‐transfer emissions. The thin films demonstrate high sensitivity and rapid response times, highlighting their potential for use in medical imaging, security screening, and environmental monitoring. Subsequently, Lin and Wang demonstrated the innovative use of a thorium‐based MOFs as a host matrix for a photoresponsive guest molecule, enabling highly sensitive and selective X‐ray dosimetry through radical‐induced photochromism [258]. The incorporation of thorium led to a record‐low detection limit of 0.047 Gy among photochromic sensors. The exclusive stability and electronic properties of thorium facilitate radical generation and stabilization within the framework. Furthermore, the same group presented a novel thorium‐based nanocluster, Th‐101, which exhibits unprecedented dual fluorochromic and piezochromic behavior under ionizing radiation, enabling highly sensitive and wide‐range (0–15 kGy) colorimetric X‐ray dosimetry via RGB‐based readout [256]. The incorporation of thorium, a high‐Z actinide, significantly enhances X‐ray attenuation and facilitates efficient energy conversion, while the unique electronic structure and strong ligand bonding ensure exceptional radiolytic stability (up to 6 MGy). The integration of Th‐101 into a compact optoelectronic device allows for real‐time, on‐site dose quantification. Fabrication of such materials into thin films would enable the development of flexible, large‐area, and wearable radiation sensors, with significant potential for applications in medical diagnostics, radiation safety, and high‐dose industrial processing.

Schmidt and coworker presented the innovative development of a thorium‐based MOF, Th‐INA‐1, constructed from isonicotinic acid, which exhibits exceptional radiation resistance—withstanding doses up to 6 MGy of β‐ or γ‐irradiation—while maintaining structural integrity [259]. Its high thorium content (∼47 wt.%) and unique hexanuclear cluster topology contribute to superior stability and selective crystallization capability in the presence of competing fission products. The incorporation of high‐Z thorium significantly enhances X‐ray attenuation, making such materials highly suitable for radiation detection applications. The coating of Th‐INA‐1 as thin film would offer great potential for use in nuclear environments, medical imaging, and portable dosimetry devices. Lin's group also presented various thorium‐based cluster materials, which uniquely integrates reversible X‐ray‐induced radiochromism and efficient radiation shielding within a single platform—a dual‐functionality rarely achieved in previous studies [58, 260]. Its millimeter‐scale single crystals enable direct visualization of X‐ray penetration and exhibit attenuation efficiency comparable to leaded glass.

In 2025, a flexible 2D uranium‐based MOF (SCU‐334) is reported by Wang and coworkers, exhibiting significantly enhanced radiation resistance for X‐ray imaging applications [261]. Its innovation lies in the use of a flexible organic linker and abundant hydrogen bonds, which dissipate radiation energy efficiently and enable self‐healing, retaining over 90% luminescence after 50 Gy irradiation—outperforming prior rigid uranium MOFs. Uranium is essential due to its high atomic number and intrinsic luminescence, providing strong X‐ray absorption and efficient scintillation. Processing into flexible films facilitates integration into large‐area, bendable detectors, enabling high‐resolution (11 lp/mm) X‐ray imaging with excellent mechanical and environmental stability for practical applications. A highly sensitive luminescent dosimeter was also produced by a novel uranyl‐based MOFs (U‐OX‐PIP) for ultralow‐dose detection of both UV and X‐ray radiation [262]. Its innovation lies in the radical‐mediated fluorescence quenching mechanism, enabled by radiation‐induced C–O• species within the robust uranyl–oxalate framework, achieving a record‐low detection limit of 8.61 × 10^−^^5^ Gy.

### 4.2 Optical Responsive Materials and Devices

The development of optical responsive materials, particularly those exhibiting photoelectric conversion and stimulus‐dependent luminescence, has garnered significant attention for applications in sensing, imaging, and optoelectronics. Among these, f‐MOFs offer unique opportunities due to their tunable electronic structures and rich photophysical behaviors. While Ln‐MOFs have been extensively explored for their luminescent properties—such as sharp line‐like emissions and long lifetimes—An‐MOFs present a largely untapped resource with complementary and often superior characteristics. Notably, An‐MOFs exhibit distinctive photoelectronic properties, such as enhanced electron‐phonon coupling, broad and intense ligand‐to‐metal charge transfer (LMCT) bands, and efficient energy transfer pathways, which are seldom observed in their lanthanide analogues [17]. The integration of actinide and lanthanide centers within f‐MOF thin films further enables synergistic functionalities. Such advanced architectures not only broaden the scope of stimuli‐responsive behaviors but also pave the way for novel optoelectronic devices, including high‐sensitivity phototransistors and ratiometric fluorescent thermometers. Thus, the targeted development of f‐MOF thin films—especially those incorporating actinides—holds great promise for achieving unprecedented performance in optical sensing and light‐driven applications.

Wang and coworkers demonstrated a heterobimetallic actinide‐lanthanide organic framework (SCU‐UEu‐2) that achieves near‐unity energy transfer from UO_2_^2^^+^ to Eu^3^^+^, yielding a record photoluminescence quantum yield of 92.68% [263]. The incorporation of uranium significantly enhances the luminescence efficiency of EuMOFs, which due to the unique photophysical properties of uranyl stemming from the intrinsic HOMO‐LUMO transition. Such An/Ln‐MOFs combine strong stopping power with high luminescence efficiency, ideal for radiation detection. Processing into thin films would facilitate device integration, improve response speed, and enable flexible composite designs for advanced scintillation and radiation protection applications.

Shustova and coworkers explores the development of heterometallic An‐MOFs with photoresponsive properties [184]. These MOFs incorporate both actinides and transition metals, allowing for dynamic and static tuning of their electronic properties. The research focuses on the synthesis, structural characterization, and investigation of their optoelectronic properties, revealing the potential for these materials in advanced electronic applications. One significant highlight of the study is the application of these MOFs in MOF‐based field‐effect transistors (FETs). The thin films of the MOFs exhibit remarkable optoelectronic properties, such as tunable band gaps and photoresponsiveness, making them suitable for use in FETs. These properties enable the MOFs to function effectively as semiconducting materials in electronic devices, opening new avenues for the design and development of advanced MOF‐based optoelectronic systems

**FIGURE 13:**
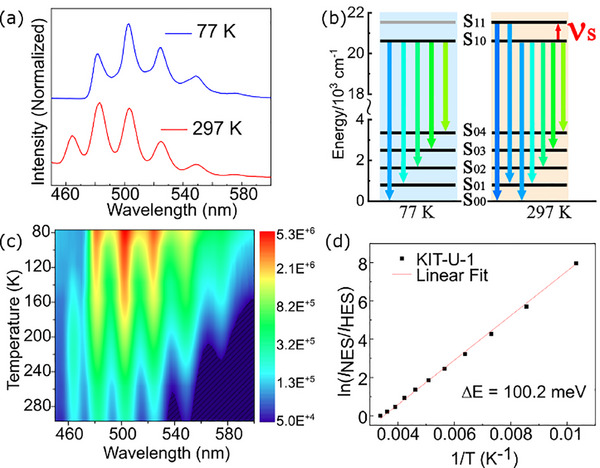
(a) Emission spectra of KIT‐U‐1 thin film at 77 K (blue) and 297 K (red); (b) schematic illustration of the emission process in KIT‐U‐1 thin film at 77 and 297 K; (c) Two‐dimensional map of the temperature‐dependent photoluminescent spectra of KIT‐U‐1 thin film; (d) Plotting of the natural logarithm of photoluminescent intensity ratio between the normal excited state and the first hot excited state against reciprocal temperature for KIT‐U‐1, R_2_ = 0.998. Reproduced with permission [170]. Copyright 2024, Wiley‐VCH.

Chen et al. explored the innovative use of uranium‐based MOF thin films [170]. These films were synthesized using a layer‐by‐layer technique, resulting in consistent, crystalline, and oriented SURMOFs. One of the standout features of these uranium‐based MOF thin films is their phonon‐assisted temperature‐dependent photoluminescence, which manifests in two unusual thermal‐activated emission bands, also known as “hot‐bands.” As presented in Figure 13, a significant application highlighted in the study is the development of a single‐metal ratiometric optical thermometer. This device leverages the unique thermal sensitivity of the uranium‐based MOF thin films to measure temperature with high precision. The exceptional thermal sensitivity and stability of these films make them particularly suitable for optical thermometry, providing a new avenue for temperature sensing technologies in various scientific and industrial applications.

### 4.3 Potential of An‐MOF Thin Films in Emerging Application

Beyond their well‐documented roles in X‐ray scintillation and optical responsive materials, An‐MOFs have emerged as promising materials in several other cutting‐edge applications, particularly in selective adsorption, gas separation, and heterogeneous catalysis [18, 29]. Compared to actinide inorganic materials, actinide MOFs (An‐MOFs) provide a greater diversity of well‐defined and highly tunable coordination environments [264]. At the same time, the inherent stability of the MOF framework helps to protect the performance‐defining properties of the actinide ions against degradation induced by changing external conditions [265]. The developments of An‐MOFs have drawn considerable interest, owing to the distinctive physicochemical properties of actinide ions—including their large ionic radii, accessible multiple oxidation states, and abundant coordination environments—which, when integrated with the structural modularity and high surface area of MOFs, lead to unique functionalities not easily achieved with typical transition metal or lanthanide‐based frameworks. For instance, the presence of highly charged actinide nodes and open metal sites promotes strong and selective interactions with small molecules such as water, iodine or xenon/krypton mixtures, making An‐MOFs exceptional candidates for gas separation and nuclear waste treatment. Likewise, in heterogeneous catalysis, the multivalent nature and Lewis acidity of actinide centers facilitate a range of chemical transformations, including C–H activation, CO_2_ conversion, and redox reactions, with enhanced selectivity and stability.

These disciplines continue to generate substantive theoretical and practical innovations. Wang and coworkers presented a novel reaction‐induced preorganization strategy to construct a heterobimetallic An‐MOF, SCU‐16‐U, with thorium and uranium precisely arranged within a single crystalline architecture [266]. The in situ oxidation of formate to carbonate creates a uranyl‐specific coordination site, enabling efficient incorporation of hexavalent uranium. This heterobimetallic An‐MOF exhibits enhanced bifunctional catalytic properties, leveraging the complementary Lewis acidity and photocatalytic activity of distinct actinide centers, thereby highlighting the potential of multimetallic An‐MOFs in advanced catalytic applications. Another work presented a series of uranyl‐organic coordination polymers constructed using a semirigid benzimidazole‐based carboxylate ligand, which enables precise tuning of coordination geometry and enhances both photocatalytic activity and structural stability [267]. The materials featured hydroxyl‐bridged dinuclear uranyl centers and high porosity, exhibits exceptional visible‐light absorption, efficient charge separation, and superior catalytic performance in the oxidative coupling of benzylamines.

Recent years An‐MOFs also exhibited exceptional potential for the adsorption of small molecules due to their high porosity, tunable structures, and abundant active sites [268, 269]. For instance, thorium MOFs demonstrate remarkable iodine capture capacity and luminescent sensing for iodate, while uranyl phosphonate frameworks show high stability and efficient uptake of americium and toxic gases like SO_2_ and NH_3_. Their unique coordination environments and structural robustness make An‐MOFs promising materials for radionuclide sequestration and environmental remediation.

The fabrication of such An‐MOFs into thin films represents a critical step toward their practical implementation, enabling improved mass transport, higher accessibility to active sites, and easier integration into device architectures. Thin‐film configurations not only maximize the exposure of functional actinide sites but also facilitate the construction of composite membranes and catalytic interfaces, thereby opening new pathways for advanced separation technologies and flow‐reactor catalysis [270, 271]. Thus, the transition from bulk powders to thin films is essential for harnessing the full application potential of An‐MOFs in these emerging fields.

In summary of this section, An‐MOF thin films constitute an emerging frontier in materials science, with profound potential for applications in X‐ray scintillation, optical thermometry, and radiation detection. The synergistic integration of actinides, distinctive electronic and spectroscopic properties with the structural tunability of MOFs renders these materials uniquely suited for advancing radiation‐related technologies. This review comprehensively surveys recent progress in An‐MOF thin films, emphasizing their synthetic strategies, functional properties, and applications in these specialized domains. Despite these promising developments, research on An‐MOFs remains in its nascent stages, with current efforts primarily focused on structural exploration and preliminary application studies. Consequently, significant opportunities persist for further investigation into their design, performance optimization, and technological integration.

## 5 Conclusions and Outlook

f‐MOF thin films have emerged as a versatile platform for harnessing the distinctive electronic, magnetic, photophysical, and catalytic properties of lanthanide and actinide ions, offering opportunities that extend well beyond those of conventional f‐element compounds. Within this class, lanthanide‐based MOF thin films represent the more established and extensively studied systems. A growing body of research has demonstrated their remarkable potential across a range of advanced applications, including sensing, photonics, and optoelectronics, owing to their unique combination of tunable optical behavior, electronic versatility, and well‐defined, porous crystalline structure. Recent studies on Ln‐MOF thin films have increasingly focused on the development of tailored ligands, architecturally diverse frameworks, and advanced thin‐film deposition techniques to enable precise responses to specific external stimuli and experimental conditions. By leveraging the distinctive spatial configurations of ligands, the stable resonance energy levels of lanthanide ions, and the diverse pore structures within Ln‐MOFs, researchers have explored new frontiers in high‐sensitivity and high‐selectivity optical or electrical sensing devices for gas and liquid phases. Additionally, the development of Ln‐MOF thin films for anti‐counterfeiting technologies, chiral luminescence, and other advanced applications holds significant promise. A particularly promising direction lies in the integration of Ln‐MOF thin films into wearable devices and biomimetic skin. The flexibility, tunable porosity, and responsive nature of these materials make them ideal candidates for next‐generation wearable sensors, energy‐efficient displays, and adaptive artificial skin systems. These applications could revolutionize fields such as healthcare, robotics, and human‐machine interfaces, offering unprecedented functionality and performance.

In contrast to lanthanides, actinides have so far received less attention. An‐MOF thin films remain in their nascent stages, presenting both challenges and opportunities for future research. Advances in An‐MOF crystal and powder studies are expanding the library of candidate materials, which will provide critical insights for the design and optimization of An‐MOF thin films. Given the radioactive nature of actinides, developing mild synthetic approaches and stabilizing their structural frameworks is of paramount importance. Drawing lessons from the evolution of Ln‐MOF thin films, establishing reliable and scalable fabrication techniques for high‐quality An‐MOF thin films will be a crucial first step. The radioluminescence and nuclear interactions of actinides enable An‐MOF thin films to selectively capture and detect radionuclides, offering potential for safer nuclear fuel processing, waste management, and radiation detection. Furthermore, the distinctive 5f electronic configurations of actinides endow An‐MOFs with exceptional functionalities in photoresponse and potential catalytic applications. The development of An‐MOF thin films will not only enhance the material's safety and practicality but also pave the way for their integration into functional devices. Future efforts should focus on overcoming synthetic challenges, elucidating structure‐property relationships, and exploring their potential in both nuclear and non‐nuclear applications.

The potential convergence of lanthanide and actinide MOF thin film research offers a unique opportunity to address complex challenges in energy, environmental science, and advanced materials. By combining the optical and electronic advantages of lanthanides with the nuclear and radiation‐related properties of actinides, hybrid MOF systems could be developed for multifunctional applications. For example, dual‐lanthanide‐actinide MOF thin films could be designed for simultaneous optical sensing and radiation detection, enabling real‐time monitoring in hazardous environments.

An especially desirable property to incorporate into framework materials and into f‐MOFs is electrical conductivity. Since earlier reports on MOFs based on metals such as Fe and Co have been retracted [272], Ln‐MOFs based on La and Nd were the first framework compounds to demonstrate metallic behavior [273]. For device integration, however, the development of f‐MOF thin films with proper electrical contacts—such as standard four‐point probe configurations—is essential. To date, Cu_3_(HHTP)_2_ SURMOFs fabricated via the layer‐by‐layer method [22]. are the only MOF thin films in which metallic conductivity has been clearly established. Extending such investigations to Ln‐based SURMOFs would be highly desirable, as this could open up a new field of applications for electrically conductive f‐MOF thin films.

In conclusion, the future of lanthanide‐ and actinide‐based MOF thin films is bright, with numerous high‐impact applications on the horizon. Continued interdisciplinary collaboration, coupled with advancements in synthesis, characterization, and theoretical modeling, will be essential to unlock the full potential of these materials. As the field matures, these thin films are poised to play a transformative role in addressing global challenges related to energy, security, and environmental sustainability.

## Funding

This work was financially supported by the National Natural Science Foundation of China (Grant No: 22405044).

## Conflicts of Interest

The authors declare no conflicts of interest.

## Data Availability

The authors have nothing to report.
